# PerfuPul—A Versatile Perfusable Platform to Assess Permeability and Barrier Function of Air Exposed Pulmonary Epithelia

**DOI:** 10.3389/fbioe.2021.743236

**Published:** 2021-10-06

**Authors:** Patrick Carius, Aurélie Dubois, Morvarid Ajdarirad, Arbel Artzy-Schnirman, Josué Sznitman, Nicole Schneider-Daum, Claus-Michael Lehr

**Affiliations:** ^1^ Department of Drug Delivery (DDEL), Helmholtz-Institute for Pharmaceutical Research Saarland (HIPS), Helmholtz Centre for Infection Research (HZI), Saarbrücken, Germany; ^2^ Department of Pharmacy, Biopharmaceutics and Pharmaceutical Technology, Saarland University, Saarbrücken, Germany; ^3^ Department of Biomedical Engineering, Technion—Israel Institute of Technology, Haifa, Israel

**Keywords:** air-liquid interface (ALI), permeability, perfusion, transepithelial electrical resistance (TEER), aerosol deposition, drug testing, pulmonary epithelia

## Abstract

Complex *in vitro* models, especially those based on human cells and tissues, may successfully reduce or even replace animal models within pre-clinical development of orally inhaled drug products. Microfluidic lung-on-chips are regarded as especially promising models since they allow the culture of lung specific cell types under physiological stimuli including perfusion and air-liquid interface (ALI) conditions within a precisely controlled *in vitro* environment. Currently, though, such models are not available to a broad user community given their need for sophisticated microfabrication techniques. They further require systematic comparison to well-based filter supports, in analogy to traditional Transwells®. We here present a versatile perfusable platform that combines the advantages of well-based filter supports with the benefits of perfusion, to assess barrier permeability of and aerosol deposition on ALI cultured pulmonary epithelial cells. The platform as well as the required technical accessories can be reproduced *via* a detailed step-by-step protocol and implemented in typical bio-/pharmaceutical laboratories without specific expertise in microfabrication methods nor the need to buy costly specialized equipment. Calu-3 cells cultured under liquid covered conditions (LCC) inside the platform showed similar development of transepithelial electrical resistance (TEER) over a period of 14 days as cells cultured on a traditional Transwell^®^. By using a customized deposition chamber, fluorescein sodium was nebulized *via* a clinically relevant Aerogen^®^ Solo nebulizer onto Calu-3 cells cultured under ALI conditions within the platform. This not only allowed to analyze the transport of fluorescein sodium after ALI deposition under perfusion, but also to compare it to transport under traditional static conditions.

## Introduction

Animal models have undoubtedly been essential for the development of oral inhalation drug products, especially for demonstrating safety as well as, at least for some diseases, also efficacy in preclinical research. But it must be realized that, already in healthy state, animal models hardly reflect the human respiratory tract with regard to the administration and deposition of aerosolized medicines. While forced inhalation or tracheobronchial instillation may still allow to draw some conclusion about pulmonary toxicity, the problem becomes more challenging for efficacy studies. This is especially true for inhalable anti-infective drugs, where the available animal models fail to adequately replicate how such diseases affect the human respiratory tract (Lorenz et al., 2016). This can be attributed to evident species-species variations between humans and model organisms (e.g., in lung anatomy, airway histology, cellular composition of epithelial and sub epithelial compartments) that amongst other reasons eventually slow down the development of orally inhaled drug products (Barnes et al., 2015; Artzy-Schnirman et al., 2019a; Jimenez-Valdes et al., 2020).

In contrast to animal models, complex *in vitro* models, especially when human based, allow to focus on key elements of underlying (patho-) physiological conditions as observed in the clinic and to model such conditions within a controlled *in vitro* environment (Carius et al., 2021). Because at present no such predictive *in vitro* models are available yet, their technological development and subsequent validation represent important and demanding scientific tasks (European Commission Joint Research Centre, 2021). Pulmonary *in vitro* models thereby profited from utilizing well-based permeable growth supports (e.g., Transwell^®^) as a cell culture environment, because these substrates most importantly enable the establishment of air-liquid interface (ALI) conditions as well as polarized differentiation of pulmonary epithelial cells (Lacroix et al., 2018). The easy accessibility to the apical as well as to the basolateral compartment further supports aerosol deposition and/or permeability studies, along with the biophysical measurement of barrier properties *via* transepithelial electrical resistance (TEER). Various simple pulmonary *in vitro* models, usually consisting of bronchial or alveolar epithelial cell monocultures, but also complex models comprising pulmonary epi- and/or endothelial cells in co-culture with other cell types like immune cells (e.g., dendritic cells, macrophages or neutrophils) or fibroblasts have been extensively reviewed (Gordon et al., 2015; Hittinger et al., 2015; Hittinger et al., 2017; Ehrmann et al., 2020).

In moving beyond simple Transwell^®^ cultures that are mainly limited by static culture conditions, organ-on-chip systems have advanced miniaturized biomimetic devices that allow the *in vitro* culture of human cells under physiological conditions similar to *in vivo*, including continuous perfusion and mechanical deflection (Tenenbaum-Katan et al., 2018). Lung-on-chip devices may moreover replicate the characteristics of specific regions of the lung (e.g., cell composition, exposure to air (flow), or breathing dynamics) and comprise tissue relevant cell types. Among the earliest efforts, Nalayanda et al. (2009) described the ALI culture of the ATII-like carcinoma cell line A549 under low flow conditions (0.35 μl/min) in a PDMS-based lung-on-chip for up to 3 weeks. Following the pioneering work of Huh et al. (2010), that introduced an alveolar-capillary model able to co-culture pulmonary epithelial cells under ALI and flow conditions together with endothelial cells, both stretched by cyclic mechanical strain, the field rapidly advanced. Punde et al. (2015) followed the concept of a dynamic Transwell®-like device, thereby showing that the implementation of dynamic flow created a concentration gradient that effectively guided the transmigration of fibrocytes in an inflammation lung model. An anatomically inspired true-scale acini-on-chip allowed the investigation of the immune response of alveolar epithelial cells in co-culture with differentiated THP-1 macrophage-like cells to nebulized lipopolysaccharide (LPS) as a surrogate for bacterial infections (Artzy-Schnirman et al., 2019a). Recently, the same group used a branching airway-on-chip platform to realistically mimic the transport and deposition of aerosolized particulate matter and study its cytotoxic effect on normal human bronchial epithelial (NHBE) cells (Elias-Kirma et al., 2020). Other devices worth mentioning enable the displacement of a flexible membrane in a diaphragm-like motion (Stucki et al., 2015) in combination with perfusion (Cei et al., 2020) as well as the membrane-free culture of airway smooth muscle cells in co-culture with epithelial cells embedded within a hydrogel (Humayun et al., 2018) or the culture of human alveolar epithelial cells in a gelatin methacryloyl hydrogel resembling alveoli-like hemispheres in a breathing lung-on-chip (Huang et al., 2021).

Despite such advances, the lung-on-chip models described above are technically cumbersome to manufacture and unfortunately need either sophisticated microfabrication techniques, stemming from academic labs with a high level of bioengineering expertise and equipment, or are too costly to be introduced in a standard bio-/pharmaceutical laboratory. Inspired by these devices, we here provide the blueprint as well as a technical proof of principal study of a novel versatile perfusable platform to assess permeability and barrier function of air exposed pulmonary epithelia (PerfuPul), using the bronchial carcinoma cell line Calu-3. Furthermore, to mimic chronic infections of epithelial cells and to enable repetitive treatment of these cultures *in vitro* with aerosolized drug products, survival times should be spanning multiple days, ideally even weeks. Hence, we hypothesized that constant perfusion of cell culture medium could not only prolong the survival time of such complex infected co-culture models, by removal of bacterial toxins and virulence factors, but also accelerate and enhance cellular differentiation, as supported by others (Chandorkar et al., 2017). To this end, we identified ALI conditions, aerosol deposition, TEER measurements and especially perfusion as a physiological relevant clearance mechanism as needed prerequisites for the intended infection studies. Our platform fulfills the technical requirements for the intended infection models and can easily be reproduced in any bio-/pharmaceutical laboratory with a moderate time as well as financial investment.

## Materials and Methods

### Design and Fabrication of the Perfusable Platform

**FIGURE 1:**
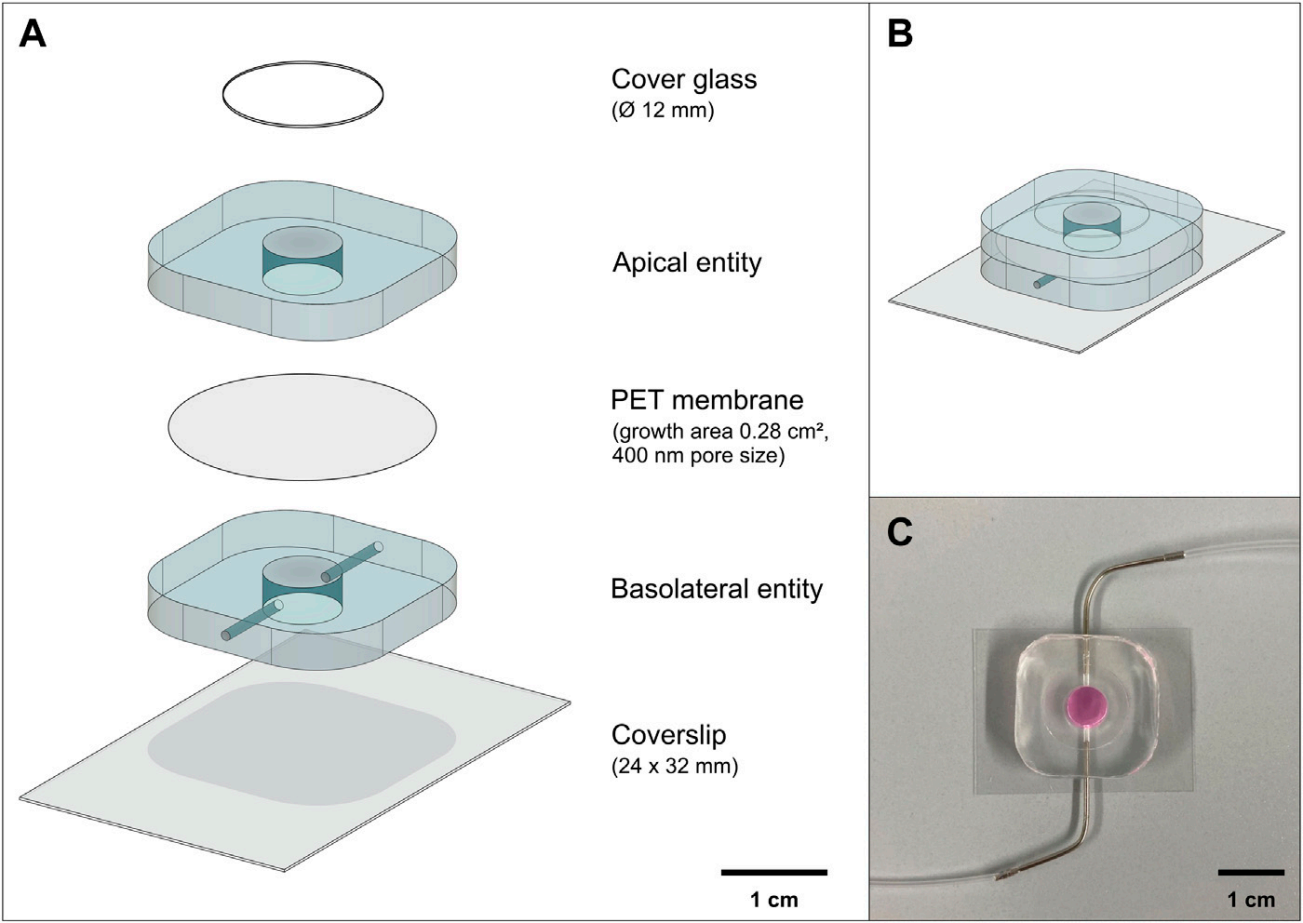
Overview of the perfusable platform “PerfuPul”. **(A)** Exploded computer-aided drawing (CAD) view of the perfusable platform made from PDMS. (B+C) The assembled platform **(B)** can be closed with a cover glass during cell culture **(C)**.

The perfusable platform consists of two well-based entities (apical and basolateral) separated *via* a permeable membrane and mounted on a glass coverslip (Figure 1A). The apical entity (chamber volume: 85 µl) can be closed with a removable cover glass (cover glass round 12 mm; Carl Roth GmbH, P231.1). Channels embedded in the basolateral entity (chamber volume: 85 µl) allow access to the basolateral chamber, to connect flexible 22ga polyethylene tubing (Instech, BTPE-50) or to insert a pair of electrodes for TEER measurements (Figures 1B,C, 3A,B).

**FIGURE 2:**
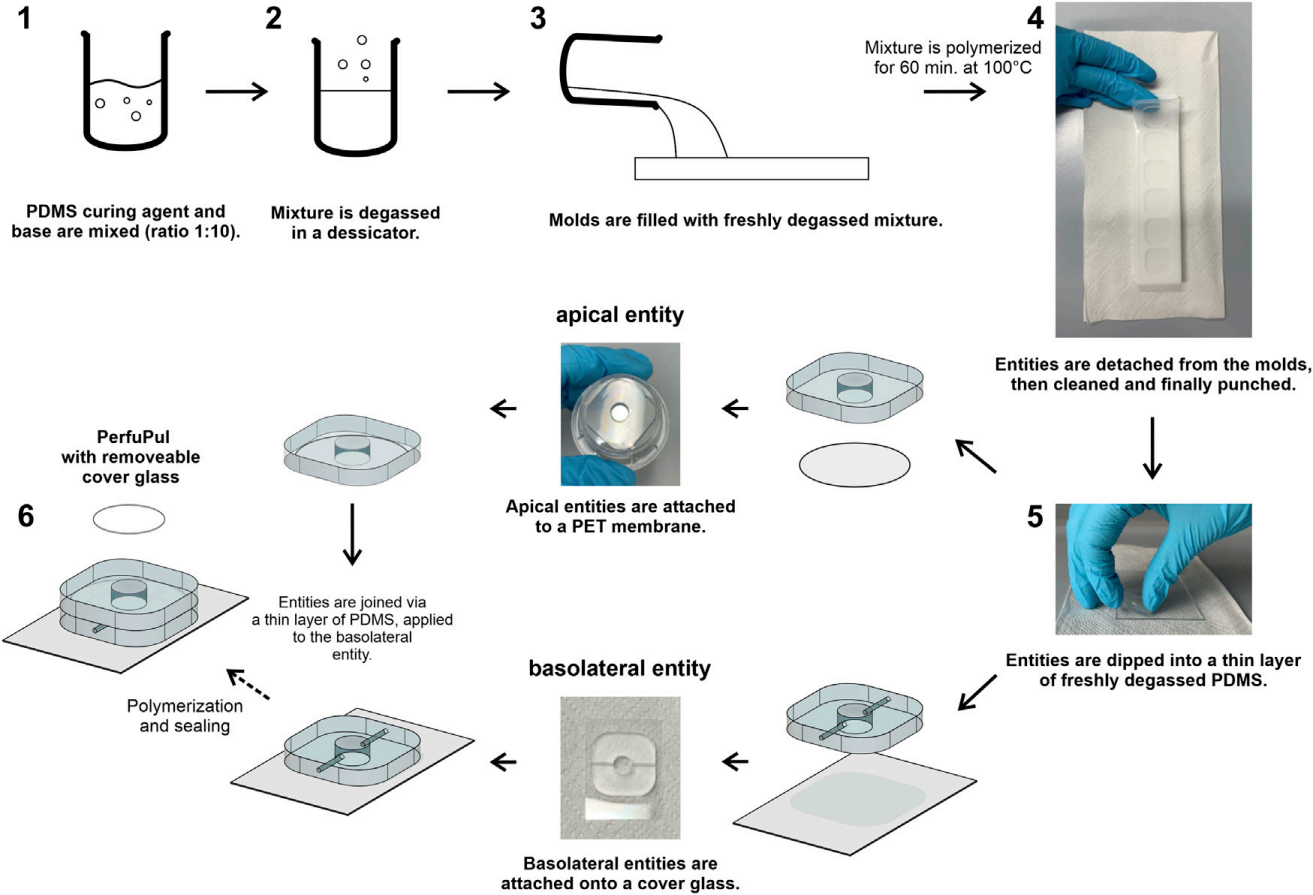
Essential steps to reproduce the perfusable platform “PerfuPul”. Steps 1 to 6 sequentially depict the essential steps to reproduce “PerfuPul”. Step 5 is performed equally for two distinct manufacturing steps, one for the apical entity and a separate one for the basolateral entity. The apical and the basolateral entity are finally combined in the last step (6). A detailed step-by-step protocol is provided in the Supplementary information.

The production of the perfusable platform is based on an adapted version of the protocol described by Artzy-Schnirman et al. (2019b), where the main steps of the production process are shown in Figure 2. Detailed engineering drawings and all steps that are needed to reproduce the platform are depicted in the Supplementary information (Supplementary Figures S3–S25; Supplementary Table S1). Engineering drawings and technical figures were created using Fusion360™ (Autodesk^®^; version 2.0.7402) under an education license. In short, two separate molds serve as a negative for the castings. The castings yield 6 apical or 6 basolateral entities respectively (Supplementary Figure S5). They were machined at the workshop of Saarland University (Saarbrücken, Germany) from polytetrafluoroethylene (PTFE). In case of the basolateral mold, the negatives for the channels were formed by insertion of 6 needles (Sterican size 12; B. Braun, 4657624). Polydimethylsiloxane (PDMS) (Sylgard 184 Elastomer Kit; Dow Corning, 1673921) was mixed with curing agent [10:1 (v/v) ratio; base/curing agent] and degassed using a desiccator. After pouring degassed PDMS onto the molds, the molds were degassed additionally and baked for 60 min at 100°C. The cured castings were peeled off from the molds, the entities were excised out of the castings and centrally punched using a biopsy punch (6 mm; Kai medical, BP-60F) to generate the wells. Apical entities were attached to polyethylenterephthalat (PET) membranes (0.4 µm pore size; Corning, 3450) and basolateral entities to a 24 × 32 mm coverslip (coverslip 24 × 32 mm; Carl Roth GmbH, H 877) *via* a “stamping” method (Chueh et al., 2007). In brief, degassed liquid PDMS [10:1 (v/v) ratio] is poured on a microscopy slide (microscope slide 76 × 52 × 1 mm; Paul Marienfeld GmbH & Co. KG, 1,100,420) that was previously cleaned, first with water followed by 100% isopropanol and then dried. After that, PDMS was spin-coated (3,000 rpm; acceleration 100 rpm/s for 60 s), resulting in a thin layer. Entities were carefully applied on to the thin layer of PDMS and subsequently attached, either to PET membranes (apical entities) or to microscopy slides (basolateral entities). After degassing, the processed entities were baked for 15 min at 100°C. Basolateral entities were combined with the apical entities repeating this process, resulting in the final perfusable platform (Figure 2, step 5-6).

### Cell Culture

#### General Cell Culture

Calu-3 cells (HTB-55™; ATCC) passages 35 to 55 were cultured in a T75-flask supplemented with 13 ml fresh minimum essential medium (MEM) containing Earle’s salts and l-glutamine (11095080), 1% non-essential amino acids (NEAA, 40035), 1 mM sodium pyruvate (11360070), 100 U/ml penicillin, 100 μg/ml streptomycin (15140122) and 10% fetal calf serum (FCS) (all Gibco™, Thermo Fisher Scientific Inc.) every two to 3 days. Cells were maintained at 37°C in a humidified atmosphere containing 5% CO_2_. When reaching 80–90% confluency, cells were detached with Trypsin-EDTA 0.05% (Gibco™, Thermo Fisher Scientific Inc.) and then seeded into a new T75-flask (2 × 10^6^ cells per flask) and/or used for the experiments detailed in the following paragraphs. All solutions were pre-warmed to 37°C before use.

#### Transwell^®^ Experiments

0.33 × 10^5^ Calu-3 cells were seeded in 200 µl MEM including all supplements per apical compartment of a Transwell^®^ insert (0.33 cm^2^; 400 nm pore size; Corning, 3,470) (1 × 10^5^ cells/cm^2^). The basolateral compartment was supplemented with 800 µl MEM including all supplements. Every two to 3 days used medium was aspirated from the basolateral compartment first and then from the apical compartment. Fresh MEM including all supplements was supplemented first in the apical compartment (200 µl) followed by the basolateral compartment (800 µl).

#### Perfusable Platform

Before cell culture, all platforms including tubing (Supplementary Figure S24) were transferred to one Petri dish (Petri dish 145 × 20 mm; Greiner Bio-One, 6052085) per platform and decontaminated for 30 min on each side (apical side facing up first, then basolateral side facing up) *via* UV light (254 nm) within a safety cabinet. Filling of the perfusable platform was achieved by manually flushing 800 µl of MEM including all supplements carefully through the basolateral compartment using a bubble-free 1 ml syringe (Injekt®-F SOLO; B. Braun, TZ-2180), leaving 200 µl of medium in the syringe. 0.28 × 10^5^ Calu-3 cells were seeded apically in a volume of 85 µl MEM including all supplements (1 × 10^5^ cells/cm^2^) and the perfusable platform was closed with an autoclaved cover glass. Every two to 3 days medium exchange was performed by carefully removing the cover glass with a sterile forceps, then aspirating the used medium from the apical compartment. After that the basolateral compartment was flushed with 800 µl from a bubble-free 1 ml syringe filled with MEM including all supplements leaving 200 µl medium in the syringe. The outlet of the basolateral compartment was closed with an autoclaved tubing clamp (Th.Geyer, 6200838). In a final step, 85 µl of MEM including all supplements was added to the apical compartment to restore LCC and the perfusable platform was closed apically with an autoclaved cover glass. The same procedure was performed for ALI conditions, with the exception, that when cells were confluent on day 7 or 8 of culture all medium in the apical compartment was aspirated. Additionally, the apical compartment of perfusable platforms containing Calu-3 cells grown under ALI conditions were washed with 85 µl pre-warmed PBS on days of medium exchange. If not stated otherwise, the perfusable platforms were always closed with an autoclaved cover glass and placed in a 145 mm diameter Petri dish at 37°C in a humidified atmosphere containing 5% CO_2_.

#### Confocal Laser Scanning Microscopy

##### Immunofluorescence Staining

For the representative immunofluorescence staining only inserts with TEER values > 500 Ω*cm^2^ were selected. After washing the apical and basolateral entity with pre-warmed PBS (apical: 85 μl, basolateral: 800 µl) all liquid was flushed out the basolateral compartment by a bolus injection of air. Cells were fixated with 85 µl of 4% paraformaldehyde (in PBS) for 10 min at room temperature (RT) from apical only. Permeabilization and blocking of unspecific epitopes was performed with blocking buffer [1% BSA (Bovine Serum Albumin heat shock fraction; Sigma-Aldrich, A9647-50G), 0.05% Saponin (Saponin Quillaja sp.; Sigma-Aldrich, S4521-10G) in PBS (w/w/v)] for 1 h at RT. Primary antibodies against tight junction proteins Occludin (monoclonal antibody, Thermo Fisher Scientific, Cat# 33-1500, RRID:AB_2533101) and ZO-1 (monoclonal antibody, BD Biosciences, Cat# 610966, RRID:AB_398279) were both diluted [1:200 (v/v)] in blocking buffer and incubated for 12 h at 4°C. The secondary antibody [1:2000 (v/v) in blocking buffer] was incubated for 1 h at RT. Nuclei were stained with DAPI [1 μg/ml in PBS (v/v)] for 30 min at RT. All steps were performed with a volume of 85 µl and the perfusable platforms were washed in between steps with PBS at RT three times. After staining, the apical compartment including the membrane was carefully detached from the basolateral entity using a forceps, by slowly inserting a scalpel underneath the membrane but not touching the growth area. Briefly, the growth area of the membrane was cut from the basolateral side of the membrane as a squared shape, roughly 1 × 1 cm in size, using a scalpel, mounted on a microscope slide (Superfrost; Menzel, AAAA000080##32E) and embedded with fluorescence mounting medium (DAKO, S3023). Samples were always kept moist by careful addition of PBS during the cutting and mounting procedure.

#### Image Acquisition and Processing

Z-stacks were acquired with an inverted confocal laser scanning microscope (TCS SP8, Leica) equipped with a ×25 water objective, using a zoom of 1, a resolution of 1,024 × 1,024, and a scan speed of 200 Hz. Maximum projections were equally created for all images with FIJI/Image J (Schindelin et al., 2012) and further processed using the BIOP Channel tools plugin (https://c4science.ch/w/bioimaging_and_optics_platform_biop/image-processing/imagej_tools/ijab-biop_channel_tools/).

### TEER Measurements

#### Transwell Insert

In case of Calu-3 cultured in Transwell^®^ inserts under LCC, TEER was measured with a chopstick electrode connected to a Volt-Ohm-meter (STX2 and EVOM 2; World Precision instruments) according to the manufacturer’s instructions. During the time of the measurement the Transwell^®^ plate was placed on a heating plate (37°C). Ohmic resistance values were corrected for the area of the Transwell^®^ insert (0.33 cm^2^) as well as the related value of a blank and reported as Ω*cm^2^. If not described differently, all cultures were fed after TEER measurement.

#### Perfusable Platform

##### Custom Electrode Fabrication

Two Ag/AgCl electrodes were created by following the procedure described by Rootare and Powers (1977), with the exception that the silver disk from the method described was replaced by a silver wire with an outer diameter of 0.5 mm (silver wire 0.5 mm diameter; neoLab, 2-3309) for each electrode. Pre-coated electrodes are also commercially available. The two Ag/AgCl electrodes (V1 and V2) and two additional pieces of silver wire (I1 and I2) were cut to a length of 15 mm, soldered to the stranded wires of a RJ14 (6P4C) telephone cable as described in Supplementary Figure S3 and insulated with a shrinkage tube per strand. The custom-made electrode was equilibrated in 100 mM KCl overnight connected to a switched off EVOM 2 in “Ohm” mode before its first use, in order to stabilize its electrical potential. After this, the electrode was stored and handled in the same way as the STX2 electrode according to the manufacturer’s instructions. To validate the custom-made electrode against the STX2 electrode, the TEER of the same Transwell^®^ with Calu-3 cells (day 16–day 19) grown under LCC was first measured with the STX2 electrode and then with the custom-made electrode. For measuring TEER in the Transwell^®^ with the custom-made electrode the electrode pair I2/V1 was placed in the basolateral compartment and I1/V2 in the apical compartment.

##### TEER Measurements in the Perfusable Platform

**FIGURE 3:**
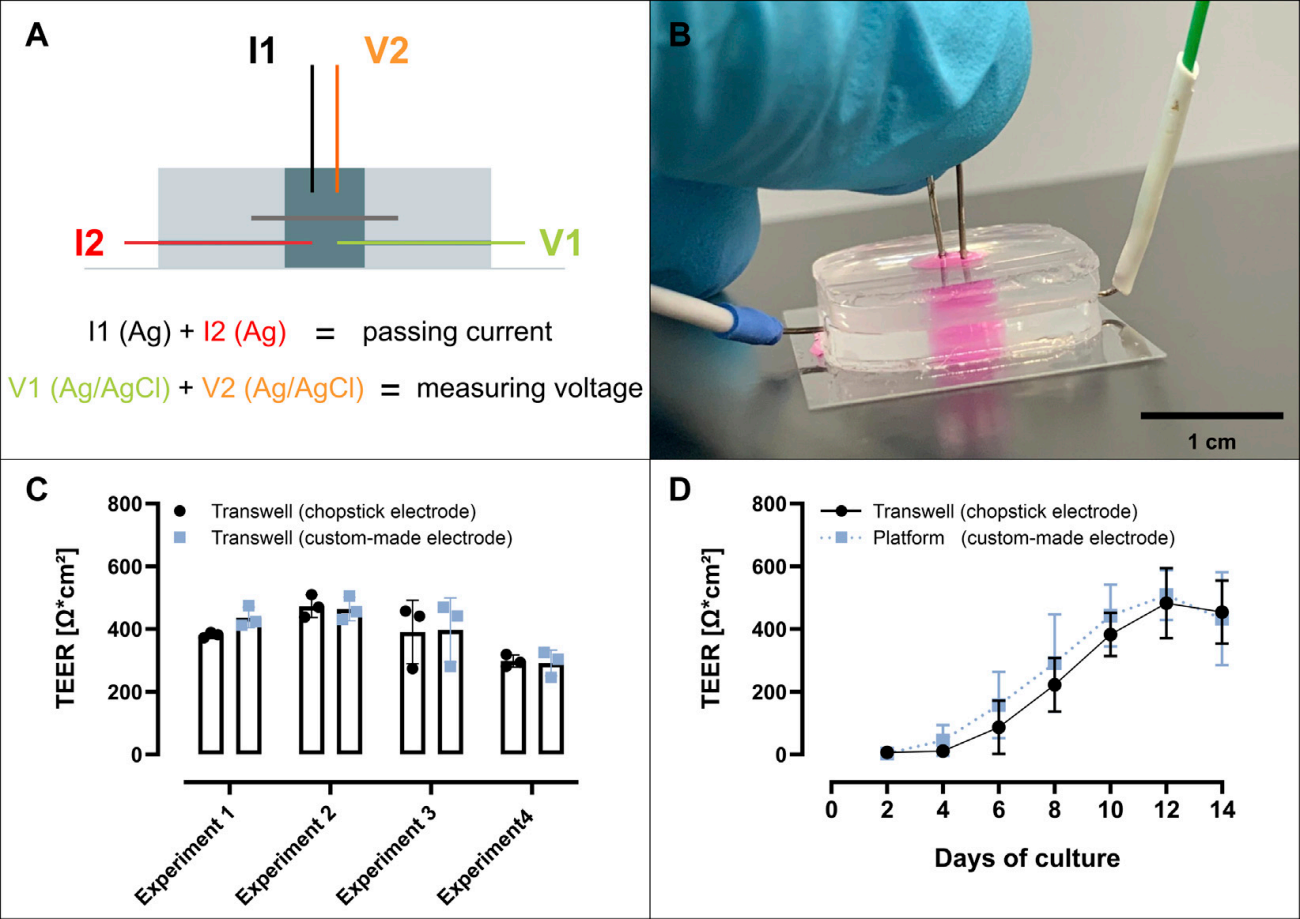
TEER measurement inside the perfusable platform. (A + B) Working principle of the custom-made electrode **(A)** for TEER measurement inside the perfusable platform **(B)**. **(C)** The custom-made electrode was validated against the commercial chopstick electrode (STX-2), by measuring Transwells from 4 separate experiments each containing 14 day old Calu-3 cells grown at LCC. Transwells were first measured with the chopstick followed by the custom-made electrode. **(D)** No difference was observed between the TEER measurements from Calu-3 cells grown under LCC in the perfusable platform or on Transwell, over the course of 14 days n = 9 (d14 Platform n = 8) out of 3 independent experiments. Data represent mean ± S.D.

The custom-made electrode was first soaked in 70% isopropanol [in sterile MilliQ water (v/v)] for 5 min and then dried before each measurement. Briefly, TEER measurements of Calu-3 cells grown under LCC were performed as described for the Transwell^®^, by placing the electrode pair I2/V1 in the basolateral compartment and the electrode pair I1/V2 in the apical compartment (Figure 3A). In the case of Calu-3 cells grown under ALI conditions, LCC were reestablished by following the procedure described for medium exchange. After an incubation time of 1 h TEER measurements were performed as described for LCC. The TEER value for each platform measured on day 3 was used as a blank for the LCC cultures. In order to not disturb the development of an ALI, for all experiments under ALI conditions the blank was set to 178 Ω*cm^2^ for these experiments. This was the upper deviation from the mean of all blanks measured for the LCC cultures at day 3 (155 Ω*cm^2^ ± 23 Ω*cm^2^; n = 6). Ohmic resistance values were corrected for the area of the perfusable platform (0.28 cm^2^) as well as for the related value of a blank and reported as Ω*cm^2^.

### Aerosol Deposition

#### Deposition Chamber Design and Fabrication

The custom-made deposition chamber was machined at the workshop of Saarland University (Saarbrücken, Germany) from a polyoxymethylene (POM) rod (Supplementary Figure S4). The design was modified based on the device published by Horstmann et al. (2021) in order to fit the perfusable platform. The chamber was designed in such a way that the wider inlet of the cylindrical device fits tightly against an Aeroneb^®^ Lab nebulizer (Aerogen^®^, Galway, Ireland) and the narrow outlet seals against the apical compartment of the perfusable platform (Figure 5A). In the upper to middle part of the deposition chamber, the inner diameter of the chamber matches the inner diameter of the nebulizer and then conically tapers towards the outlet, where a nozzle protrudes 2 mm from the main body of the deposition chamber. The nozzle is inserted into the apical entity of the perfusable platform and leaves a distance of 1 mm towards the apical surface area, ensuring that it will not interfere with any cells. The distance between the vibrating mesh of the nebulizer and the cell layer is ∼50 mm and was chosen due to handling reasons. It can be extended by lenghthening the upper to middle part of the custom-made deposition chamber as needed. Aerosol loss is avoided by insertion of a sealing ring (22 mm inner diameter; 2 mm cord size) in the nebulizer-fitting cavity of the deposition chamber and a tight fit of the nozzle inside the perfusable platform.

#### Deposition Protocol

The Aeroneb^®^ Lab nebulizer as well as the deposition chamber were disinfected with 70% isopropanol and allowed to dry before the experiments. Before each use, the Aeroneb^®^ Lab nebulizer (standard VMAD, 2.5–4.0 µm droplet diameter) connected to an Aerogen^®^ USB controller (both Aerogen^®^, Galway, Ireland) was tested for a constant liquid output rate which was not allowed to differ more than 10% from 0.5 ml/min. Therefore 200 µl of sterile PBS was nebulized and the time needed to nebulize all liquid was taken. For aerosol deposition experiments the Aeroneb^®^ Lab nebulizer was connected to the custom-made deposition chamber and inserted into the open apical cavity of the perfusable platform. If not stated otherwise, 20 µl of a fluorescein sodium solution (1 mg/ml in PBS) were allowed to nebulize completely and the generated aerosol settled for 1 min before the nebulizer and the deposition chamber were removed from the perfusable platform. After aerosol deposition the perfusable platform was immediately closed with a sterile cover glass.

For the determination of the deposited dose (Figure 5B), single apical entities of the perfusable platform were directly attached to a 24 × 32 mm microscopy slide using the “stamping” method described for the fabrication of the perfusable platform. 20 µl of a fluorescein sodium solution (1 mg/ml in PBS) were nebulized into 30 µl of PBS which were previously pipetted into each apical entity and served as a surrogate for the cell layer. Fluorescence intensity of nebulized fluorescein sodium was measured in 96-well plates at 485 nm excitation and 530 nm emission wavelength with a plate reader (Infinite M200 Pro; Tecan Trading AG) and the deposited dose was calculated from a standard curve. This was done for 5 separate entities. The deposition efficiency was reported as the percentage of the measured dose from the invested dose before nebulization.

### Transport Studies

Before each transport experiment, TEER values of Calu-3 cells cultured between day 17 and day 19 were measured while cells remained under LCC to ensure barrier integrity (before). Samples were only used for transport experiments when TEER values reached >300 Ω*cm^2^ before the transport study in accordance with Ehrhardt et al. (2002).

#### Transwell^®^ System

Calu-3 cells were washed once with pre-warmed Hanks’ Balanced Salt Solution (HBSS) with CaCl_2_ as well as MgCl_2_ (HBSS (1x); Gibco™, Thermo Fisher Scientific Inc., 14025050) and then equilibrated in HBSS (200 µl apical; 800 µl basolateral) for 1 h (1 h after switch). After measuring TEER, HBSS was aspirated from both, the apical and basolateral compartment. 200 μl fluorescein sodium solution (2.5 μg/ml in HBSS) were added apically (donor) and 800 µl HBSS were added to the basolateral compartment (acceptor). From the same solutions 200 μl each were transferred into a 96-well plate to determine the starting concentrations for each compartment. All steps were performed on a heating plate at 37°C. Afterwards, the Transwell^®^ plates were placed on a MTS orbital shaker (150 rpm; IKA, Germany) in the incubator and 200 μl samples were taken every 1 h for a total of 7 h, from the basolateral compartment only. 200 µl sampled at time points were immediately replenished with 200 µl pre-warmed HBSS. TEER was measured 30 min after the last sample was taken (after 8 h), 200 µl from the apical as well as the basolateral compartment were sampled to determine the end concentrations and all samples were measured with a plate reader in a 96-well plate at 485 nm excitation and 530 nm emission wavelength. The concentration of fluorescein sodium in each sample was calculated using a calibration curve of defined concentrations of fluorescein sodium in HBSS.

#### Perfusable Platform

Calu-3 cells were washed once with pre-warmed HBSS, by repeating the procedures described for medium exchange. After 1 h of equilibration TEER was measured again (1 h after switch). After connecting a fresh set of tubing including a new syringe filled with pre-warmed HBSS, the basolateral compartment was filled bubble free, while the HBSS from the equilibration step remained in the apical compartment. Immediately after the basolateral compartment was filled and closed with a tubing clamp (receiver), the HBSS in the apical compartment was aspirated and 85 μl fluorescein sodium solution (2.5 μg/ml in HBSS) was added apically (donor). Shortly after, the syringe that was connected to the basolateral compartment was placed into a syringe pump (Harvard industries, PHD Ultra) and 80 μl samples were taken every 1 h for a total of 7 h, while the perfusable platform was placed on an orbital shaker (150 rpm). The flow rate of the syringe pump was set to 1 ml/min in order to sample 80 µl in a short period of time from the basolateral compartment while additionally preventing the sampling maneuver from exerting too much pressure on the cell layer. TEER was measured 30 min after the last sample was taken (after 8 h) and 80 µl from the apical as well as the basolateral compartment were sampled to determine end concentrations.

Transport studies under ALI conditions in the perfusable platform followed the same procedure as transport studies under LCC with only a few exceptions. Calu-3 cells were set to ALI conditions between day 7 or 8 of culture and cultured until day 16–18. The first TEER measurement (1 h after switch) was performed 1 h after LCC was restored by the addition of HBSS to both compartments. After the first TEER measurement ALI conditions were restored again and Calu-3 cells were allowed to equilibrate for 30 min. Then 20 µl of a sterile fluorescein sodium solution (1 mg/ml in PBS) were nebulized onto the apical compartment. The apical compartment was closed with an autoclaved cover glass immediately after the aerosol settled for 1 min. The perfusable platform was placed on an orbital shaker (150 rpm) and 80 μl samples (ALI discontinuous sampling) were taken every 1 h for a total of 5 h, while shortly perfused (1 ml/min) with a syringe pump during the time of sample collection. For transport studies of ALI cultures under perfusion, a peristaltic pump (flow rate: 80 μl/min; Gilson, minipuls 3) equipped with a PharMed^®^ BPT tubing (internal diameter: 0.38 mm; Saint Gobain Performance Plastics™, 070539-04) was connected to the basolateral compartment and 80 μl samples (ALI continuous sampling) were taken every 1 h for a total of 5 h. Although the same dose of fluorescein sodium was used for the transport studies under ALI conditions and LCC, we reduced the duration of the transport studies under ALI conditions to 5 h due to an increased concentration gradient.

All samples were analyzed in the same way as described for the Transwell^®^ samples. The area under the curve (AUC; a. u.), C_max_ (ng/ml) and t_max_ (min) were determined from the cumulative concentration-time curve using GraphPad Prism^®^ 9 (GraphPad software).

#### Calculation of the Apparent Permeability Coefficient (Papp)

From the linear portion of a cumulative concentration-time curve (LCC: 240–360 min), where drug concentration in the receiver compartment did not exceed 10% of the drug concentration originally added to the donor compartment (Supplementary Figure S2) and at which no lag time was observed, the slope was calculated and divided by the area (A; cm^2^) of the growth support to get the flux of fluorescein sodium (J; ng/cm^2^*s). To obtain the Papp (cm*s-1) the following equation was applied, where c_0_ (ng/cm³) is the initial concentration in the donor compartment at the beginning of the experiment:

$$\mathrm{Papp}=\;\frac{J}{c_{0}}$$

The measured concentrations were converted into absolute masses of compound by multiplication with the acceptor volume of the perfusable platform which was 120 µl (= 85 µl for the chamber +35 µl for the connected tubing).

### Statistical Analysis

If not stated otherwise, numerical data were reported as individual values or mean values ± standard deviation (SD). 2-way ANOVA was performed not assuming sphericity and with a Šídák´s multiple comparisons test. Unpaired *t*-test was performed with Welch`s Correction. *p* values were defined as: ns: *p* > 0.5; *: *p* < 0.05; **: *p* < 0.005; ***: *p* < 0.0005. Calculations were made using GraphPad Prism^®^ 9.

## Results

### Concept of the Perfusable Platform

The perfusable platform has been designed in such a way, that it is both easy to produce and operable by non-experts. It encompasses an apical compartment, which is open to the top, and a basolateral compartment, which can be perfused *via* two lateral channels (Figure 1A). This design allows to keep experimental conditions similar to the established static Transwell^®^ systems or analogues thereof, and to generate analogous readouts. The open design of the apical entity enables aerosol deposition as well as easy access to the apical cell layer, and the lateral orientation of the in- and outlet enables the insertion of a custom-made electrode for measuring TEER. In addition, the apical entity can be closed with a sterile cover glass to protect the cell layer during cell culture. The two entities are separated *via* a PET membrane that is cut from a Transwell^®^, which ensures that composition and quality of the growth support are essentially the same, requiring minimal adaptations of the protocol. By keeping the height of the assembled perfusable platform to a minimum, cell growth can be monitored microscopically under sterile conditions, while the assembled perfusable platform (Figures 1B,C) remains in a Petri dish. This was demonstrated in a model experiment for the growth of Calu-3 cells, which were seeded on the apical side of the membrane within the apical entity of the perfusable platform under LCC (Supplementary Figure S1). After seeding, Calu-3 cells reached confluency within 7–8 days of cell culture, indicating that neither the setup nor the handling of the platform impaired reproducible cell growth.

### Analyzing Barrier Integrity Inside the Perfusable Platform

TEER measurements prove to be a non-destructive, reliable and functional tool for the assessment of barrier integrity. The increase in ohmic resistance of an *in vitro* culture grown on a permeable support thereby serves as a convenient readout to monitor the development of functional tight junctions and other cell-to-cell connections. To measure TEER in case of the Transwell^®^, the shorter leg of an Ag/AgCl chopstick electrode is inserted into the apical compartment, while the longer leg is simultaneously placed into the basolateral compartment. The electrode is then connected to an epithelial Volt-Ohm-Meter, which calculates the ohmic resistance.

The design of the perfusable platform, however, required another type of Ag/AgCl electrode to measure TEER values, since the narrow channels are incompatible with the commercial chopstick electrodes provided with a standard epithelial Volt-Ohm-Meter (EVOM 2) instrument. We decided for a custom-made electrode connected to the four cords of a RJ14 (6P4C) telephone cable as described in the methods section. This is the same type of cable used to connect the chopstick electrode to a regular EVOM 2. As depicted in Figure 3A, two cords of the electrode are passing current [I1 (Ag) + I2 (Ag)] and voltage is measured *via* the other two cords [V1 (Ag/AgCl) + V2 (Ag/AgCl)]. In order to measure TEER, I1/V2 need to be inserted into the apical compartment of the perfusable platform and I2/V1 need to be inserted into the basolateral compartment, while connected to an epithelial Volt-Ohm-Meter (Figures 3A,B).

The chopstick electrode, however, comprises a combination of Ag as well as Ag/AgCl electrodes per leg, one Ag pellet on the side of each leg that faces away from the Transwell^®^ insert (passing current) and an Ag/AgCl pellet per leg that faces the Transwell^®^ insert (measuring voltage). In order to show that the design of the custom-made electrode, which is based on individual silver wires and not on silver pellets attached to each leg, does not impair TEER measurements, the functionality of the custom-made electrode was compared to the chopstick electrode. Both measurements were conducted in the same Transwell^®^ (Figure 3C). For this, TEER values from 12 Transwells^®^ out of four experiments (3 Transwells^®^ per experiment) carrying Calu-3 cells grown for 14 days under LCC were measured. After 14 days TEER values were measured first with the chopstick electrode and then with the custom-made electrode. The mean of all TEER values determined with the custom-made electrode showed a deviation of +3% from the mean of all TEER values determined with the chopstick electrode (custom-made electrode: 398 ± 54 Ω*cm^2^; chopstick electrode 386 ± 41 Ω*cm^2^). These differences between the two electrodes were in an acceptable error range covering not more than 15 Ω*cm^2^, which suffices for the determination of TEER values during *in vitro* culture of pulmonary epithelial cells.

The development of TEER values of Calu-3 cells inside the perfusable platform was compared side-by-side with Calu-3 cells grown on Transwells^®^, under LCC over the course of 14 days (Figure 3D). As displayed in Figure 3D, TEER values developed equally in the perfusable platform as well as in the Transwell^®^ during 14 days of culture, reaching a maximum (perfusable platform: 510 ± 81 Ω*cm^2^; Transwell^®^: 484 ± 112 Ω*cm^2^) after 12 days of culture. These results indicated that the combination of the custom-made electrode and the perfusable platform could be used to reliably determine the development of TEER values in the same quality as the traditional combination of the chopstick electrode and the Transwell^®^.

**FIGURE 4:**
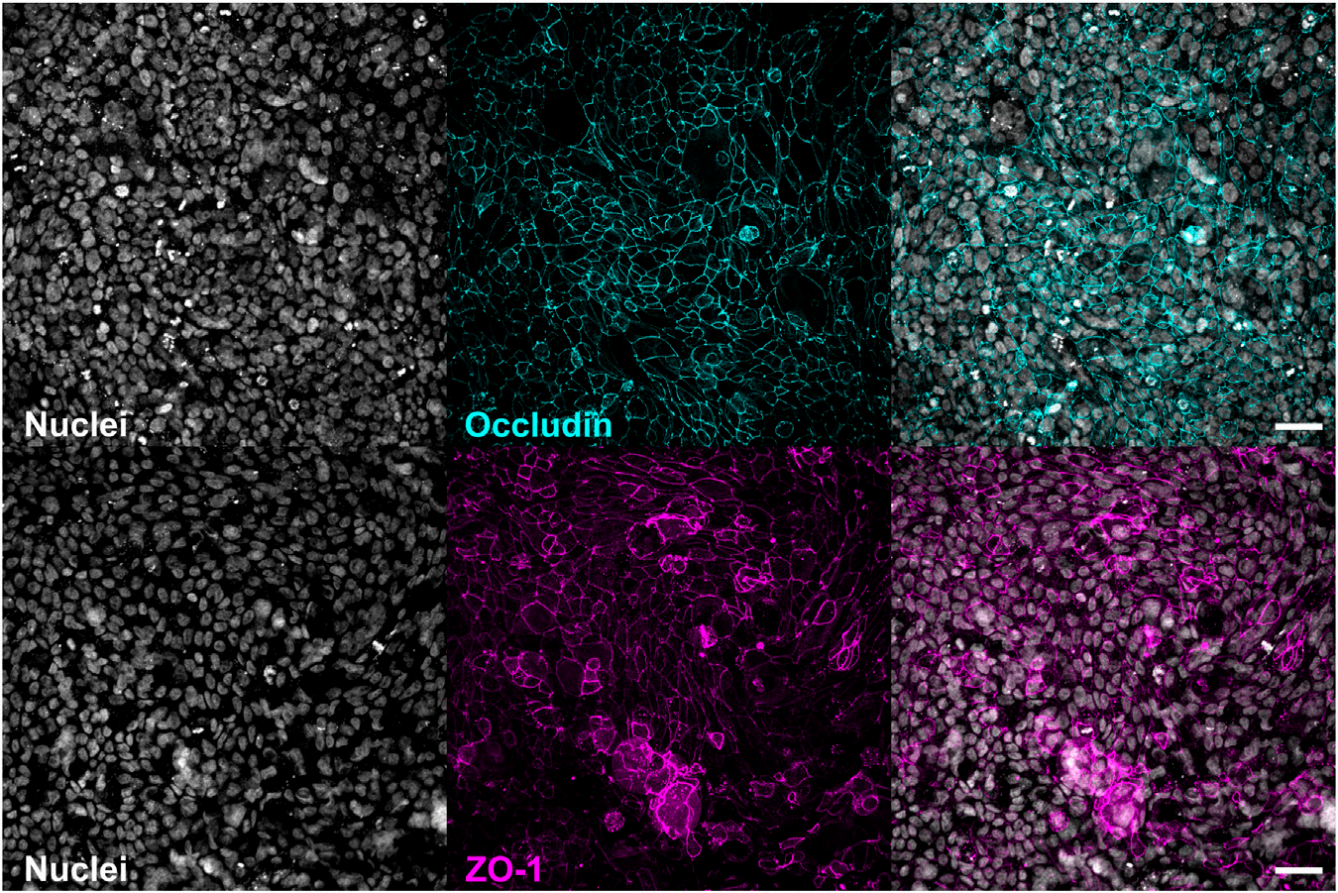
Confocal microscopy possible with the perfusable platform. Micrograph showing Calu-3 cells cultured until d16 (top) or d18 (bottom) under LCC. Calu-3 cells were fixated with 4% paraformaldehyde and stained for tight junctions Occludin (top) or ZO-1 (bottom) as well as nuclei (DAPI) to show the versatility of the perfusable platform to apply immune staining methods (scale bar: 50 μm).

Another common technique to demonstrate the integrity of a pulmonary epithelial barrier *in vitro* is the visualization of proteins that form functional tight junctions *via* fluorescent immunocytochemistry staining. Figure 4 exemplifies how such methods can be conducted within the perfusable platform in the same manner as they are applied for the Transwell^®^. As described in the methods section, Calu-3 cells were fixated and treated with the respective antibodies to visualize the tight junction forming proteins Occludin (d16) and ZO-1 (d18). Both micrographs show the development of a densely connected network representative of functional tight junctions together with a homogenously distributed cell layer indicated *via* staining of cell nuclei with DAPI.

The combination of non-destructive TEER measurements to assess barrier integrity together with the ability to perform immunocytochemistry staining after cells have been fixated demonstrates that the perfusable platform can be used for the quantitative and mechansistic characterisation of barrier function during *in vitro* culture of pulmonary epithelial cells.

### Pre-Metered Aerosol Deposition on the Perfusable Platform

**FIGURE 5:**
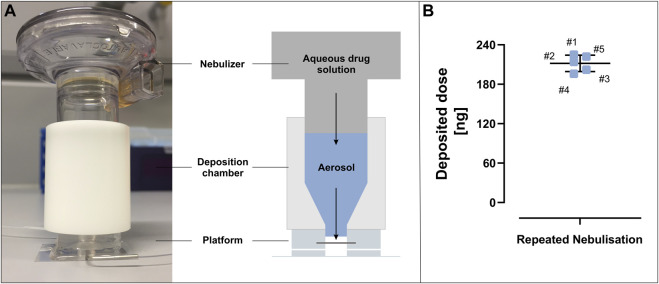
Aerosol deposition on the perfusable platform. **(A)** By attaching an Aerogen Lab nebulizer to a custom made deposition chamber, aerosols can be deposited on the perfusable platform. **(B)** Repeated nebulisation (#1–#5) of 20 µl fluorescein sodium (1 mg/ml in PBS; cloud settling time 1 min) in five different devices led to a reproducible deposited dose (mean: 212 ± 12 ng). Data represent mean ± S.D.

The *in vitro* culture of pulmonary epithelial cells under ALI conditions exposes the epithelial cell layer apically to air, which creates a physiologically relevant interface to mimic the *in vivo* situation more closely. Other than LCC, which for good reasons are standard for intestinal epithelial or blood vessel forming endothelial cells, ALI conditions also allow the controlled deposition of aerosols to the apical surface of the cell layer. Since the apical compartment of the perfusable platform can be opened, by removing the cover glass whenever needed, switching to ALI conditions and the deposition of aerosols are easily possible. In this context, we adapted the design of a recently published custom-made deposition chamber (Horstmann et al., 2021) which fits to an Aeroneb^®^ Lab vibrating mesh nebulizer (Figure 5A). The functional unit, consisting of an Aeroneb^®^ Lab nebulizer connected to an Aerogen^®^ USB controller as well as to the deposition chamber, is placed on the apical compartment of the perfusable platform. The nebulizer generates an aerosol from an aqueous drug solution through a vibrating mesh, which is released into the deposition chamber and finally settles as a mist onto the apical compartment of the perfusable platform. Reproducible deposition of pre-metered doses of an aqueous drug solution, can be achieved by controlling the concentration and/or the volume of the solution before nebulization. While increasing the settling time beyond 30-s was found to not further affect the deposited dose, a settling time of 1 min was choosen as routine to facilitate the experimental procedure (Horstmann et al., 2021).

This was demonstrated by nebulizing 20 µl of fluorescein sodium solution (1 mg/ml in PBS) onto five perfusable platforms, which comprised only the apical compartment attached to a glass slide. By keeping the settling time of the mist to 1 min after each nebulization, a delivered dose of 212 ± 12 ng could be reproducibly deposited (Figure 5B).

### Comparison of Fluorescein Sodium Transport Between the Transwell^®^ and the Perfusable Platform

To carry out comparative transport experiments on Calu-3 cells grown under LCC on Transwell^®^ versus cells grown under LCC in the perfusable platform, fluorescein sodium was used as a well-defined low-permeability marker.

**FIGURE 6:**
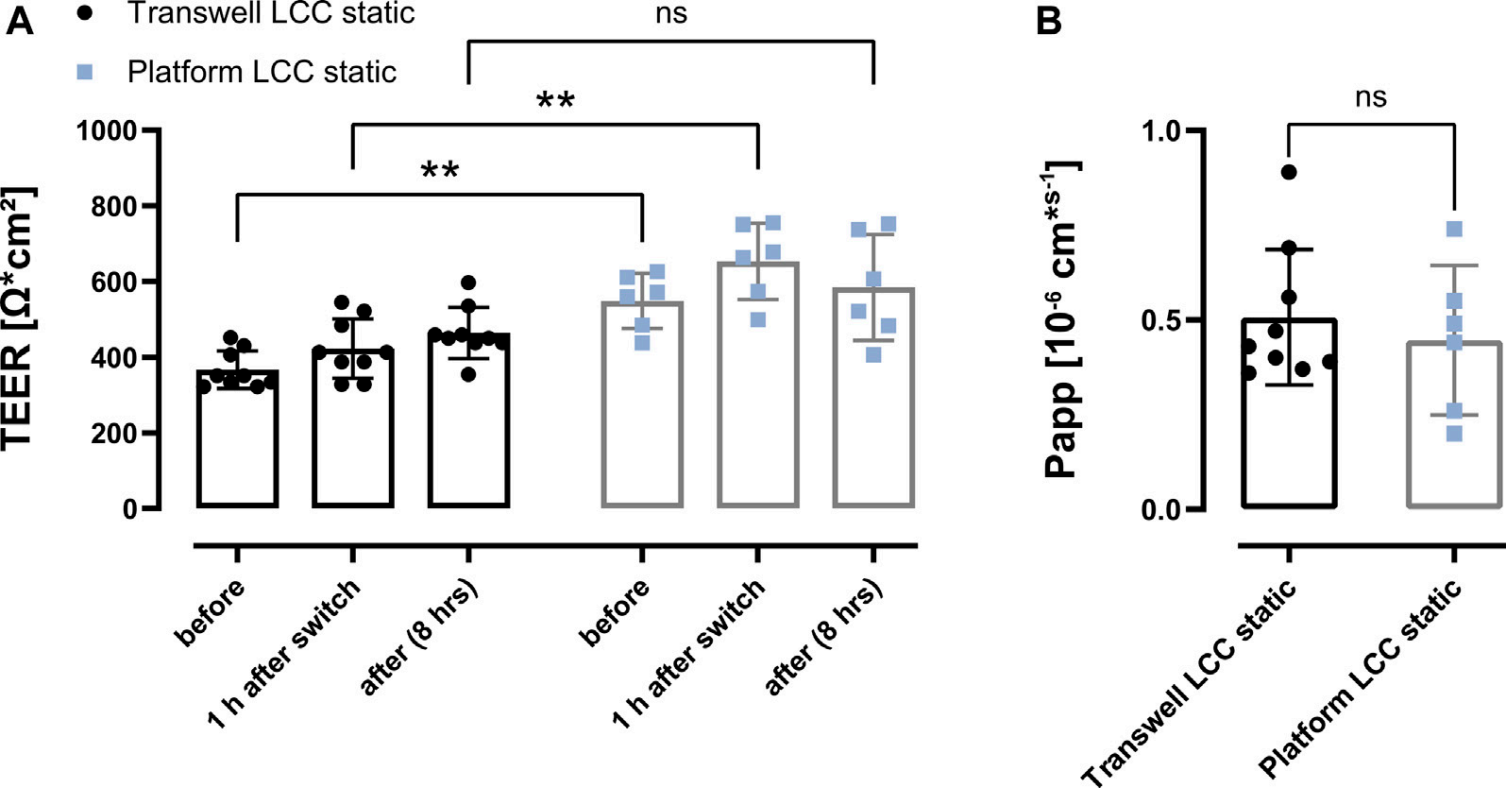
Comparison of fluorescein sodium transport between Transwell and the perfusable platform under LCC. **(A)** Transport studies were performed on Calu-3 cells (LCC: d17-d19). TEER values were measured before the experiment (before), 1 h after the incubation in transport buffer (1 h after switch), as well as after the transport study (after) and indicated a stable barrier during the transport. **(B)** Apparent permeability (Papp) of fluorescein sodium (2.5 μg/ml (dose: 500 ng on Transwell; 212 ng on platform)) applied as a solution. Papp was determined after the transport study (7 h). Data represent mean ± S.D. **(A)** 2-way ANOVA was performed not assuming sphericity and with a Šídák´s multiple comparisons test, ns: *p* > 0.5; *: *p* < 0.05; **: *p* < 0.005; **(B)** Unpaired *t*-test was performed with Welch`s Correction; Transwell LCC: n = 9, Platform LCC: n = 6 out of 3 independent experiments.

Before, during and after each transport experiment, TEER values were measured to ensure that barrier integrity was not compromised by the transport buffer or over the duration of the experiment. In case of the cells grown under ALI conditions, the first TEER measurements were performed 1 h after LCC conditions were re-established. Before the transport experiments, Calu-3 cells grown under LCC in the Transwell^®^ presented a significantly lower TEER (before, 367 ± 49 Ω*cm^2^) than Calu-3 cells grown in the perfusable platform under the same conditions (before, 548 ± 73 Ω*cm^2^) (Figure 6A). After the switch to HBSS as a transport buffer (1 h after switch) TEER values slightly increased in both the Transwell^®^ cultures (423 ± 78 Ω*cm^2^) as well as in the cultures grown in the perfusable platform (to 653 ± 100 Ω*cm^2^). After 7 h of transport experiments (Transwell^®^: 464 ± 67 Ω*cm^2^; perfusable platform: 584 ± 140 Ω*cm^2^), TEER values did not decline when compared to the condition before the transport experiments for either system, indicating that barrier properties remained intact during the course of the experiment. Without reaching statistical significance, however, it was observed that in the perfusable platform TEER values after the transport slightly decreased (−69 ± 4 Ω*cm^2^) compared to 1 h after the switch to transport buffer, whereas the TEER values of the cells cultured on Transwells^®^ showed a slight inscrease (41 ± 3 Ω*cm^2^).

The Papp values further supported the assumption of the formation of a functional diffusional barrier to fluorescein sodium within Calu-3 cell layers, which TEER value measurements already indicated (Figure 6B). The transport of fluorescein sodium over Calu-3 cell layers grown under LCC within Transwell^®^ inserts showed no significant differences indicated by the respective Papp values when compared to cell layers grown in the perfusable platform (Transwell^®^: 0.51 ± 0.18 10–6 cm*s-1; perfusable platform: 0.45 ± 0.20 10–6 cm*s-1). In addition, permeability of fluorescein sodium in a perfusable platform without any cells grown inside was increased 20-fold (Supplementary Figure S2).

This set of experiments showed that the perfusable platform performs as good as the Transwell^®^ under the experimental requirements of a transport study, in terms of reproducibility and consistency of results.

### Transport Studies Under Perfusion

**FIGURE 7:**
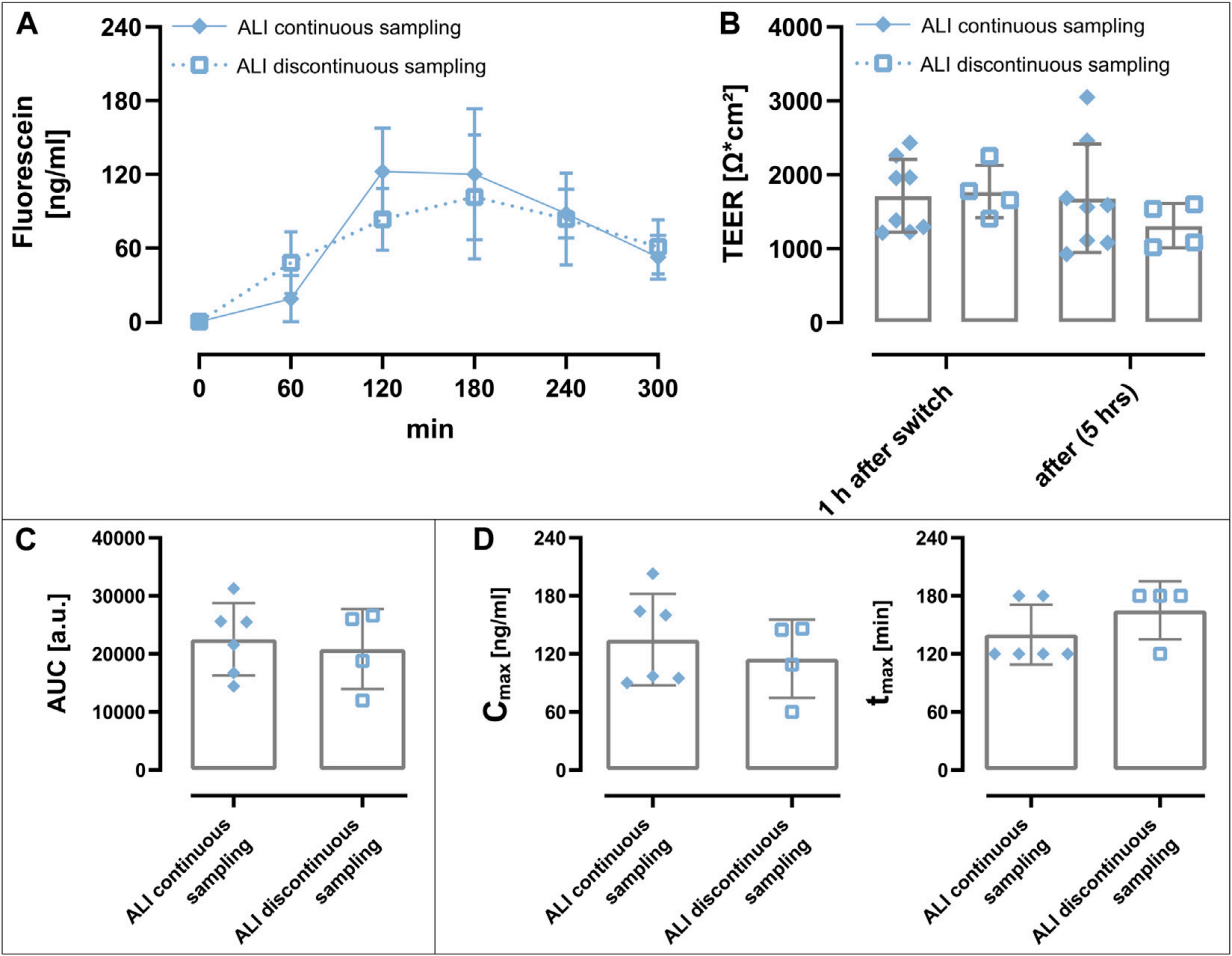
Transport studies under perfusion after ALI deposition. **(A)** Concentration time curve of nebulized fluorescein sodium (dose: 212 ng in PBS). Samples were collected every hour either sampled continuously from perfusion (80 μl/h) generated by a peristaltic pump or discontinuously (80 µl) *via* a short perfusion with a syringe pump. During the transport study, samples termed “ALI continuous sampling” were thus constantly perfused, while samples termed “ALI discontinuous sampling” were shaked at 150 rpm and only shortly perfused with a syringe pump during sample collection. 80 µl sample volume was collected for both conditions every hour. **(B)** TEER values 1 h after switching from ALI to LCC in order to determine the TEER before the transport study (1 h after switch) and at the end of the 5 h transport experiment [after (5 h)] was unchanged, indicating that epithelial barrier function was not affected by perfusion. **(C)** The area under the concentration time curve (AUC) was comparable between the two conditions. **(D)** The maximal concentrations (Cmax; left) as well as the time to reach the highest concentration (tmax, right) did not significantly differ and were rather comparable for both conditions. Data represent mean ± S.D.; ALI continuous sampling n = 6 out of 3 independent experiments, ALI discontinuous sampling n = 4 out of 4 independent experiments.

The essential advantage of the perfusable platform in comparison to the Transwell^®^ system is the option to perfuse the basolateral compartment. In order to demonstrate one of the various possibilities enabled by perfusing the acceptor compartment, we compared the transport of fluorescein sodium after nebulization under static conditions and under the influence of perfusion (Figure 7A). For the static conditions, the experimental setup for sampling was the same as for the transport studies under LCC described earlier (Figure 6), where the basolateral compartment was only subjected to a short perfusion (80 μl, ALI discontinuous sampling) from a syringe pump during sample timepoints, while the platform remained on a shaker during the transport experiment. In the case of the samples that were taken from perfusion, samples were collected as fractions (80 μl, ALI continuous sampling) from perfusion (80 μl/h) generated by a peristaltic pump. During transport experiments TEER seemed not to be affected from perfusion when compared to static conditions (Figure 7B), demonstrating that perfusion did not disturb barrier properties during a 5 h transport experiments. Neither the comparison of the different concentration time curves nor the comparison of the area under the concentration time curve (AUC) (Figure 7C), yielded a significant difference between the two conditions (ALI continuous sampling: 22,525 ± 6,248 a. u.; ALI discontinuous sampling: 20,850 ± 6,900 a. u.), indicating that similar mass transport occurred under both conditions. When comparing the mean of C_max_ (ALI continuous sampling: 135 ± 47 ng/ml; ALI discontinuous sampling: 115 ± 41 ng/ml) as well as t_max_ (ALI continuous sampling: 140 ± 31 min; ALI discontinuous sampling: 165 ± 30 min) of the individual platforms, there seems to be - without being statistically significant - at least some trend towards higher C_max_ as well as shorter t_max_ for the transport studies under perfusion (Figure 7D), but this would need further investigation.

## Discussion

In spite of their physiological advantages, perfusable transport chambers have only rarely found their way into *in vitro* models of epithelial cell culture, in particular for the lungs (Artzy-Schnirman et al., 2020). Complex designs, mostly encompassing micron-sized rectangular channels, as well as the underlying problems of impaired aerosol deposition and/or complexity of handling by non-experts, might restrict the exploitation of the full potential of lung-on-chip devices as preclinical research tools (Junaid et al., 2017; Ehrmann et al., 2020). Perfusable systems offer particular advantages for studying infectious diseases, as concentration of e.g., bacterial virulence factors can be kept at lower levels by continuous supply and dilution with fresh media. Before this can be studied in more detail, however, it is necessary to ensure to what extent data generated on novel perfusabel setups can be compared to those obtained on established Transwells^®^ under static conditions (Ainslie et al., 2019). We therefore here first describe the development as well as the characterization of a simple perfusable platform for pulmonary epithelial cell cultures at ALI conditions. This platform maintains the advantages of traditional Transwell®-based systems, but adds the possibility to implement perfusion as a prerequisite to develop longer-lasting *in vitro* models of chronic pulmonary diseases in the future. In addition, we want to share the protocol needed to produce the platform with a broad scientific community, in order to make this technology available to end-users that might not yet be familiar with microfabrication techniques.

Apart from standardized readouts to assess cell viability or cytotoxicity *in vitro*, such as the MTT or lactate dehydrogenase (LDH) assay respectively, information from TEER measurements or permeability studies appear extremely helpful for the preclinical evaluation of orally inhaled drug products (Wilson 2000; Hittinger et al., 2017). For such purposes, these models must allow ALI conditions together with the subsequent deposition of aerosols. Although pulmonary epithelial cells grown on Transwell^®^ inserts meet all of these technical specifications, they are limited to static culture conditions. Recent studies, however, demonstrated that normal human bronchial epithelial (NHBE) cells cultured under ALI conditions and simultaneous perfusion of cell culture medium showed improved barrier properties in comparison to static conditions (Chandorkar et al., 2017; Bovard et al., 2018). Perfusion is, amongst other physiological important mechanical stimuli, also recognized as a central element to be implemented in organ-on-chip systems in general to improve cellular development (Thompson et al., 2020).

The new platform thus was designed to comply with the familiar technical standards and features of Transwell^®^ inserts, but at the same time also enabling perfusion as a physiological relevant clearance mechanism not present in Transwell®-based culture plates. Major specifcations are: 1) a comparable surface area (24-well based Transwell^®^: 0.33 cm^2^; perfusable platform: 0.28 cm^2^), 2) the possibility to routinely inspect cell layers under a microscope while maintaining sterile culture conditions, 3) the option to measure TEER, and 4.) the implementation of ALI conditions as well as subsequent aerosol deposition (Figures 1, 3, 5; Supplementary Figure S1). The access to the basolateral compartment *via* a combination of a channel-based inlet connected to a flexible tubing enables the culturing of cells under static culture conditions, but also adds the possibility to perform experiments under perfusion (Figure 7).

In a side-by-side comparison using Calu-3 cells grown on regular Transwell^®^ inserts or within the perfusable platform we could show that both, the resistance readings from the two electrodes (chopstick vs. custom-made electrode) within the same Transwell^®^ insert as well as the measurements performed in the perfusable platform and on Transwell^®^ inserts under equal experimental conditions agree by good approximation (Figure 3C, D). Notably, this was irrespective of the fact that in the case of the custom electrode, when compared with the chopstick electrode within the same Transwell^®^, four separate cables needed to be arranged in each well. The custom-made Ag/AgCl electrode used by us can be connected to existing instrumentation (e.g., EVOM 2) with only little technical effort needed to solder the different silver wires to a 4-cord cable (Figure 3A), as already described for organ-on-chip systems in similar ways (Douville et al., 2010; Ferrell et al., 2010; Huh et al., 2010; Kim et al., 2012; Griep et al., 2013). Most of these studies reported significantly higher TEER values in case of the organ-on-chip systems when compared to results obtained for the same cell models cultured on Transwell^®^ inserts. However, these higher TEER values could be related to the geometry of the rectangular micron-sized channels within these organ-on-chips, that generate a non-uniformly distributed electrical current, rather than showing a biological effect (Odijk et al., 2015). The perfusable platform reported in the present paper, however, is based on a design encompassing two equally sized wells separated by a circular membrane that has a cell culture area identical to that of a 24-well Transwell^®^ insert (Figure 1A). Limiting the use of micron-sized channels only to the in- and outlet that enable access to the basolateral well, further generates similarity to the Transwell^®^.

Especially during permeability studies within the same lab, TEER value measurements using an EVOM 2 or similar direct current based Voltohmmeters are widely accepted as a valuable non-invasive readout to routinely assess barrier integrity. This holds still true while some more advanced bioelectrical methods (e.g., impedance spectroscopy) might be more precise in determining information about actual barrier integrity (Günzel et al., 2012; Odijk et al., 2015; Srinivasan et al., 2015; Henry et al., 2017).

As already indicated by the stable TEER values during the course of the 7 h transport experiments, the apparent permeability of fluorescein sodium transported under LCC also did not reveal any significant differences between the perfusable platform and the Transwell^®^ (Figure 6B). Further were the obtained Papp values in this study, in relation to the respective TEER values, consistent with reported Papp values in the literature performed under similar study conditions on Transwell^®^ inserts using Calu-3 cells (Ehrhardt et al., 2002; Fiegel et al., 2003; Grainger et al., 2006; Haghi et al., 2010). To our knowledge, these historical data from Transwells^®^ inserts have not been confirmed using any perfusable, microfluidic or lung-on-chip system so far.

To assess barrier integrity by staining for relevant cellular markers, techniques to perform immunofluorescence staining and subsequent confocal laser scanning microscopy or other imaging methods can be used with the perfusable platform in the same way as for Transwell^®^ inserts. This was demonstrated by the example of the visualization of the proteins Occludin and ZO-1 that are involved in the formation of functional tight junctions (Figure 4). For that purpose, the apical as well as basolateral entity can be separated from the membrane, independently from each other. This procedure allows to flexibly conduct staining and cell fixation methods.

One of the unique features of the perfusable platform as presented here is the possibility to reproducibly deposit pre-metered aerosols from aqueous drug solutions on pulmonary epithelial cells grown at an ALI using a clinically relevant vibrating mesh nebulizer (Aeroneb^®^ Lab). The removable cover glass, that allows to easily open and close the top of the apical compartment, thereby enables the establishment of ALI conditions after cells have been grown to confluency under LCC. The flexibility to open and close the top of the apical compartment is what creates the possibility to connect a vibrating mesh nebulizer to the perfusable platform by using the custom-made deposition chamber. The custom-made deposition chamber presented here was modified to fit the opening of the apical compartment of the perfusable platform, but mostly retains the design as well as dimensions of the device described by Horstmann et al. (2021) to be used with a single 12-well (12 mm) Transwell^®^ insert or a single 24-well. Since we extensively discussed the obtained results and characterized the deposition chamber in the work mentioned above, we limited the characterization for its use with the perfusable platform to the experiments described in Figure 5, to ensure that reproducibility of results is consistent with our previous findings. While reproducibility could be maintained in good approximation (5.6% relative standard deviation (deposition on perfusable platform) vs. 4.8% relative standard deviation [12-well Transwell^®^)], the deposition efficiency showed a 4-fold reduction [1.06 ± 0.06% (perfusable platform) vs. ∼4% (12-well Transwell^®^)]. When all parameters, such as the used nebulizer, the volume to be nebulized in the same concentration and the settling time are kept constant, the deposition efficiency in these chambers is mainly limited by the inner diameter of the outlet (Horstmann et al., 2021). The inner diameter amounts to ∼5 mm in the version for the perfusable platform and ∼11 mm for the 12-well Transwell^®^ version. A substantial amount of the aerosol mist generated by the vibrating mesh nebulizer thus deposits on the walls of the deposition chamber and therefore is not channeled through the outlet. A similar concept, also including a vibrating mesh nebulizer and a connected chamber, was recently introduced by Cei et al. (2020) in a dynamic *in vitro* stretch lung model. The outlet of the aerosol chamber in their model was about 20 mm in inner diameter and resulted in an impressive deposition efficiency of about 52% after nebulization.

Although we acknowledge the fact that a deposition efficiency of 1% from the invested dose seems relatively low, the dose delivered per surface area provides better comparability in this case. Based on an equally invested dose of 20 µg (20 µl from a 1 mg/ml solution) which would be nebulized, the delivered dose per surface area would result to ∼0.7 μg/cm^2^ (perfusable platform, 0.28 cm^2^; deposition efficiency: 1%), ∼0.8 μg/cm^2^ (12-well Transwell^®^, 1.12 cm^2^; deposition efficiency: 4.8%) or ∼2.2 μg/cm^2^ [(Cei et al., 2020), 4.67 cm^2^; deposition efficiency: 52%]. Ultimately, between different devices the dose delivered per surface area should be used for comparison instead of the isolated deposition efficiency. This considerably mitigates the major differences observed when focusing on the isolated deposition efficiency while enhancing comparability, despite the fact that the delivered dose per surface area is still about three times higher in the device of Cei et al. (2020) when compared to the perfusable platform, since less compound is deposited on the walls of the deposition chamber. The foregoing notwithstanding, although the main share of compound is deposited on the walls of the deposition chamber and not reaching the cellular layer, the quantity of compound invested to obtain meaningful *in vitro* results with the devices mentioned before is still substantially lower than that normally required for conducting *in vivo* inhalation studies (Phalen and Mendez 2009).

As demonstrated by the data of Figure 7, transport studies under static as well as perfused conditions were successfully conducted after the reproducible deposition of a pre-metered aerosol from an aqueous fluorescein sodium solution. Interestingly enough, under perfusion we only observed a negligible decrease in TEER values (−2%) after 5 h of transport after ALI deposition of fluorescein sodium under perfusion (Figure 7B) while the difference after 5 h of transport under static conditions amounted to (−26%) when comparing only the mean values. Although this difference was not statistically supported, it could suggest a positive effect of perfusion on barrier properties during the course of the transport experiment in comparison to static conditions, but would need more thorough investigation. The ability to implement perfusion into the perfusable platform, further allowed the comparison of transport of nebulized fluorescein sodium as a model compound under static conditions to transport under perfusion. Although we also could only observe a slight trend towards higher C_max_ and lower t_max_ in case of the fluorescein sodium transported under continuous perfusion, we think that if such experiments would be performed under different parameters (shorter sampling intervals, drugs with different permeation, different flow rates etc.) meaningful insights into the transport of experimental drugs intended for inhalation under physiological relevant conditions could be provided.

We are well aware that the features that make up the perfusable platform are built upon the work of other researchers active in the lung-on-chip field. A similar concept comprising a PDMS-based chip which possessed an open apical compartment, thereby allowing the establishment of ALI conditions, which was also able to provide perfusion and TEER measurements was already introduced by Nalayanda et al., in 2009. Although the authors compared their findings systematically to Transwell^®^ inserts at an ALI using the alveolar type 2-like cell line A549, they did not show the deposition of aerosols. The importance of flow in lung-on-chip systems initially demonstrated by Punde et al. (2015), was used by others to follow the concept of a flowable Transwell^®^ (Blume et al., 2015; Blume et al., 2017; Chandorkar et al., 2017; Bovard et al., 2018; Schimek et al., 2020). The problem with such systems is that the Transwell^®^ inserts where the cells are cultured during perfusion need to be taken out of the sophisticated culture devices that provide the perfusion, and are then transferred to separate culture wells or devices before TEER measurements can be performed or aerosols could be deposited. Especially for substances that are rapidly absorbed after aerosol deposition, such delays could aggravate subsequent analysis. Sophisticated aerosol deposition on cells grown at an ALI was described by Artzy-Schnirman et al. (2019a) for a morphologically inspired acinus-on-chip and also recently for a bronchial bifurcation mimic by Elias-Kirma et al. (2020). Unfortunately, these devices were not characterized in terms of TEER measurements nor in the application of perfusion. In addition, the former device needs sophisticated microfabrication during production and the latter device requires a complex set of devices for the generation of aerosols as well as the related physiologically relevant airflows. Researchers that consider implementing lung-on-chip devices for their research or just want to try out if the physiological features provided by these devices, such as perfusion, could benefit their work, are thus facing considerable difficulties, if they do not have the needed technical expertise in micro-fabrication or the financial resources to invest in commercial platforms. Although companies like Alveolix, CN bio, Emulate, Kirkstall or TissUse introduced innovative lung-on-chip devices or perfusable Transwells^®^, the material costs of the chips and the related infrastructure needed to operate them could add up to sums which are difficult to finance for academic labs (Kirkstall Ltd 2020; AlveoliX 2021; Chips - TissUse GmbH 2021; CN Bio Innovations 2021; Emulate 2021).

The perfusable platform presented here holds some limitations. The platform is based on a PET membrane, which is cut from Transwell^®^ inserts and known to be rather rigid and bio-inert in comparison to the extracellular matrix found *in vivo* (Humayun et al., 2018). The used PDMS is also known to absorb small, hydrophobic molecules (Toepke and Beebe 2006). This technical characteristic can be corrected though, by pre-equilibrating the devices or adjusting for the loss of substance (Jimenez-Valdes et al., 2020). Depending on the skill of the operator but also on the used peristaltic pump, results obtained from the perfusable platform are in the low- to mid-throughput range depending on the extent of the intended studies.

## Conclusion

The minimalistic design of the presented perfusable platform “PerfuPul” allows its production by non-experts in most lab environments without the need for specialized equipment. Furthermore, the platform combines TEER measurements, aerosol deposition as well as the implementation of perfusion as a physiological relevant clearance mechanism in a single device that matches the design as well as the quality of results known from Transwell® inserts. The open apical entity not only allows the establishment of ALI conditions but could be also utilized for working with cutting-edge techniques such as 3D bio printing. Moreover, the easy access to the basolateral compartment would permit co-culture studies, for instance, by also growing endothelial cells on the basal side of the membrane. Taking all of these factors in consideration, we are convinced that this perfusable platform will enable the development of novel pulmonary *in vitro* models especially to study long-term diseases, such as e.g., bacterial lung infections and their treatment by aerosolized drugs and nanoscale carriers therof.

## Data Availability

The raw data supporting the conclusions of this article will be made available by the authors, without undue reservation.
