# Supplementary material for: PerfuPul—A Versatile Perfusable Platform to Assess Permeability and Barrier Function of Air Exposed Pulmonary Epithelia
Source: Front Bioeng Biotechnol. 2021 Oct 6;9:743236. doi: 10.3389/fbioe.2021.743236 (PMC8526933; doi:10.3389/fbioe.2021.743236)
Supplement: Supplementary file 1 [file DataSheet1.PDF]

Supplementary Material

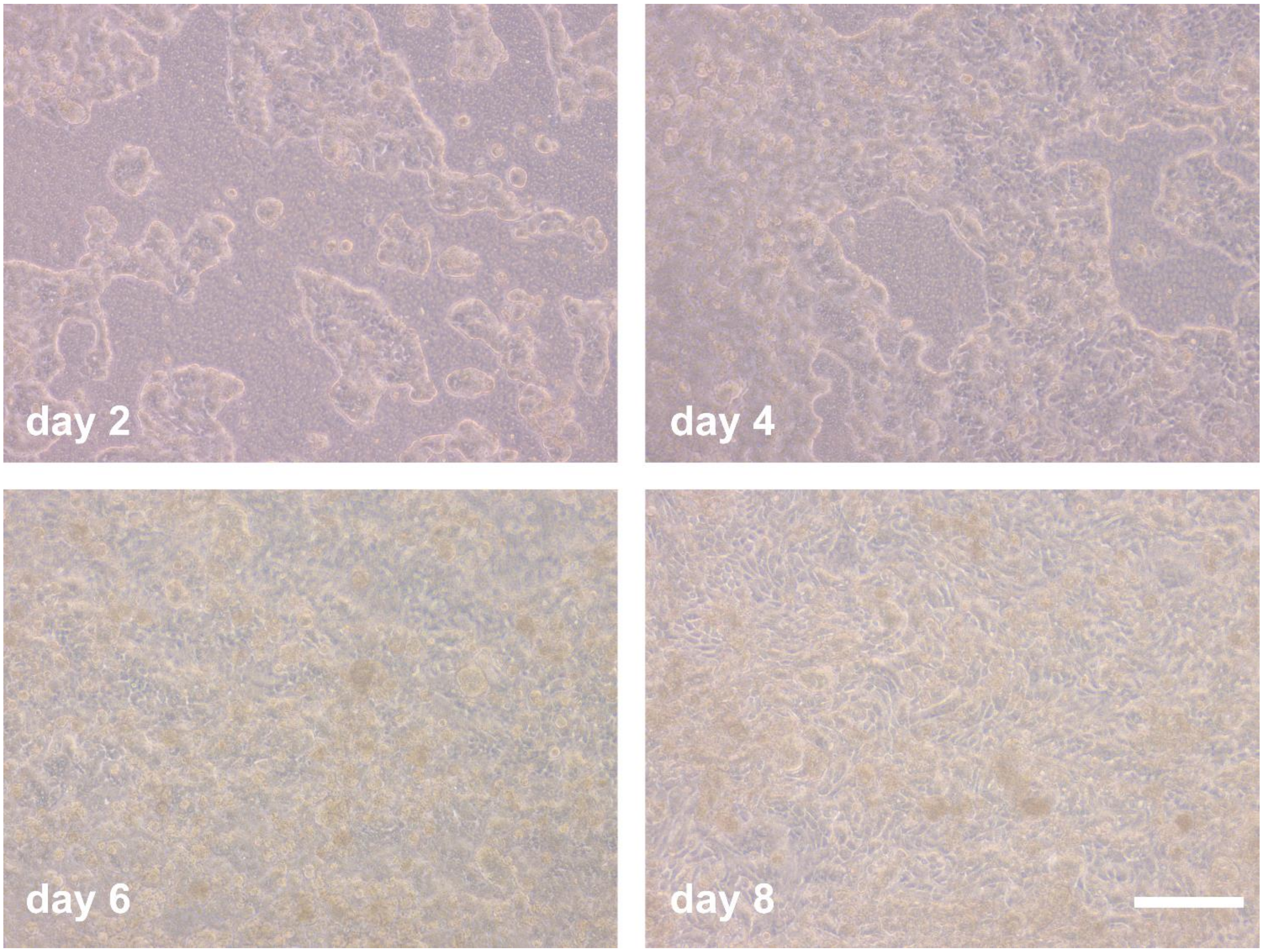

Supplementary Figure 1: Representative micrograph showing undisturbed growth of Calu-3 cells inside the perfusable platform. Calu-3 cells (10.000 cells/cm² seeded) become confluent between day 6 and day 8 of culture. Calu-3 cells were cultured under liquid covered conditions (LCC) and fed every second day. (scale bar : 200 µm)

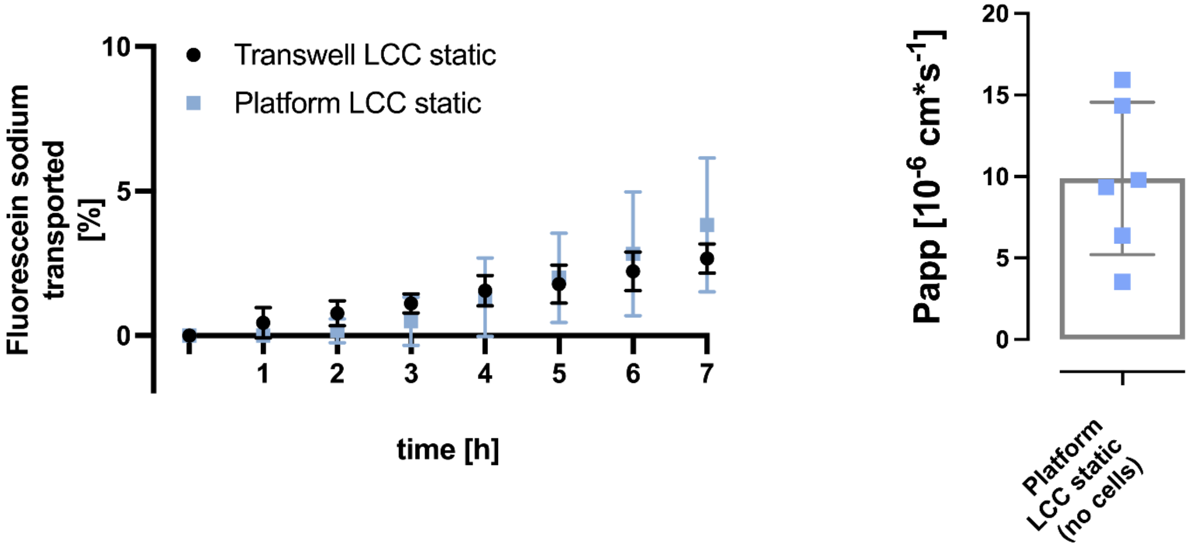

Supplementary Figure 2: Transport studies were performed on Calu-3 cells (LCC: d17-d19). **Left:** The transported Fluorescein sodium derived from the concentration within the acceptor compartment at sampling time points is cumulatively displayed in relation [%] to the concentration within the acceptor compartment at the beginning of the experiment (2.5 µg/ml, (dose: 500 ng on Transwell; 212 ng on platform)). **Right:** Apparent permeability (Papp) of Fluorescein sodium applied as a solution to perfusable platforms without any cells. Papp was determined after the transport study (100 min.). Data represent mean ± S.D.; Transwell LCC: n=9, Platform LCC & Platform LCC (no cells): n=6 out of 3 independent experiments.

The following pages contain all information needed to reproduce the platform as well as the described experiments.

Supplementary Table 1: Parts and devices needed for the production of the perfusable platform "PerfuPul" as well as other accessories.

		Name	Specifications	Item number	Supplier
**Devices needed**		**Desiccator**	not specified	not specified	not specified
		**Lab oven**	Temperature: 100°C	not specified	not specified
		**(optional) Spin coater**	should fit a 76x53 mm glass slide; min. 3000 rpm; 100 rpm/s	not specified	not specified
**Custom-made electrode**					
		**RJ14 (6P4C*) telephone cable**	*: 6 pins, 4 connected to stranded wires	not specified	not specified
		**Silver wire**	0.5 mm outer diameter	2-3309	NeoLab
		**Shrinkage tubing**	not specified	not specified	not specified
		**Soldering iron + soldering tin**	not specified	not specified	not specified
**Perfusable platform**					
	**PDMS preparation**				
		**Plastic weighing dish**	not specified	not specified	not specified
		**Polydimethylsiloxane (PDMS)**	Sylgard 184 Elastomer Kit (curing agent + base)	1673921	Dow Corning
	**Mold filling**				
		**Needle (channel negative)**	Sterican, size 12 (0.7 x 40 mm)	4657624	B. Braun
	**Entity production**				
		**Scissors**	not specified	not specified	not specified
		**6 mm Biopsy punch**	6 mm diameter	BP-60F	Kai medical
		**Adhesive tape**	Scotch® Magic™ 810 matt	7100026960	Scotch®
		**Blunt needle**	Sterican blunt, 21 G, 7/8 inch (0.8 x 22 mm)	9180109	B. Braun
	**Platform assembly**				
		**Microscopy slide (76x52 mm)**	Microscope slide 76x52x1 mm	1100420	Paul Marienfeld GmbH & Co. KG
		**Transwell insert**	24 mm, 0.4 µm pore size	3450	Corning
		**Scalpel**	not specified	not specified	not specified
		**Cover glass (24x32 mm)**	cover glass 24x32 mm	H877	Carl Roth GmbH
	**Tube assembly**				
		**Tubing**	flexible 22 G Polyethylene tubing	BTPE-50	Instech
		**2 ml reaction tube**	not specified	not specified	not specified
	**Cell culture**				
		**Petri dish**	145x20 mm	6052085	Greiner Bio-One
	**Perfusion setup**				
		**15 ml reaction tube**	not specified	not specified	(e.g. Falcon®)
		**Sterile filter cap**	from T25 cm² cell culture flask	C6481-200EA	Greiner Bio-One

1 Custom-made electrode

Materials needed: RJ14 (6P4C) telephone cable, silver wire (length; ~ 7 cm), shrinkage tubing (4 pieces), soldering iron, soldering tin

1. Cut a RJ14 cable to a length of ~ 60 cm.

2. Cut a silver wire (0.5 mm diameter; neoLab, 2-3309) into 4 pieces with a length of 15 mm per piece.

3. Coat two of the silver wire pieces with chloride using the following method:

Rootare, H. M.; Powers, J. M. (1977): Preparation of Ag/AgCl electrodes. In *Journal of biomedical materials research* 11 (4), pp. 633–635. DOI: 10.1002/jbm.820110416.

4. (**optional**) Pre-coated electrodes are also available commercially.

5. Solder one of the uncoated silver wires (Ag) to the stranded wire of the RJ 14 cable that connects to pin 3 and the other uncoated silver wire to the stranded wire that connects to pin 4. Then solder one of the coated silver (Ag/Cl) wires to the stranded wire of the RJ 14 cable that connects to pin 5 and the other coated silver wire to the stranded wire that connects to pin 6 (Supplementary Figure 3).

6. Insulate each junction where an electrode is connected to a stranded wire with a shrinkage tube.

7. Before first use, equilibrate the custom-made electrode in 100 mM KCl (in H_2O) overnight connected to a switched off EVOM2 in “Ohm” mode connected to a charger. This stabilizes the electrical potential of the electrode.

   The custom-made electrode needs to be disinfected with 70% Isopropanol (in sterile MilliQ water, (v/v)) for 5 min and then fully dried before each measurement.

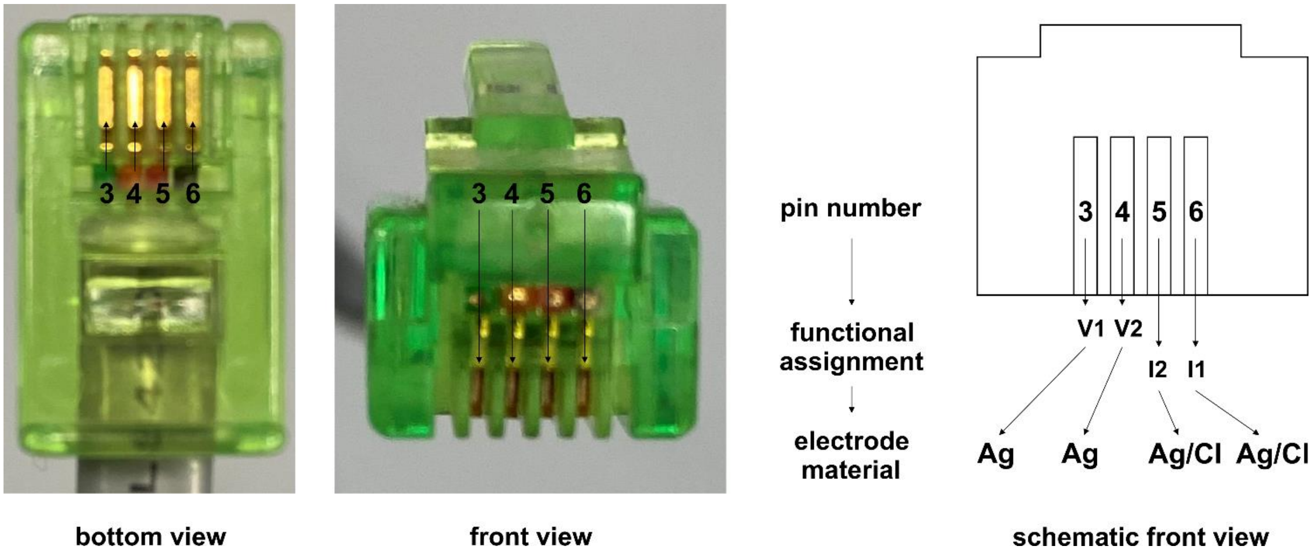

Supplementary Figure 3: Pin assignment scheme of the RJ14 (6P4C) telephone cable for the custom made electrode.

2 Deposition chamber

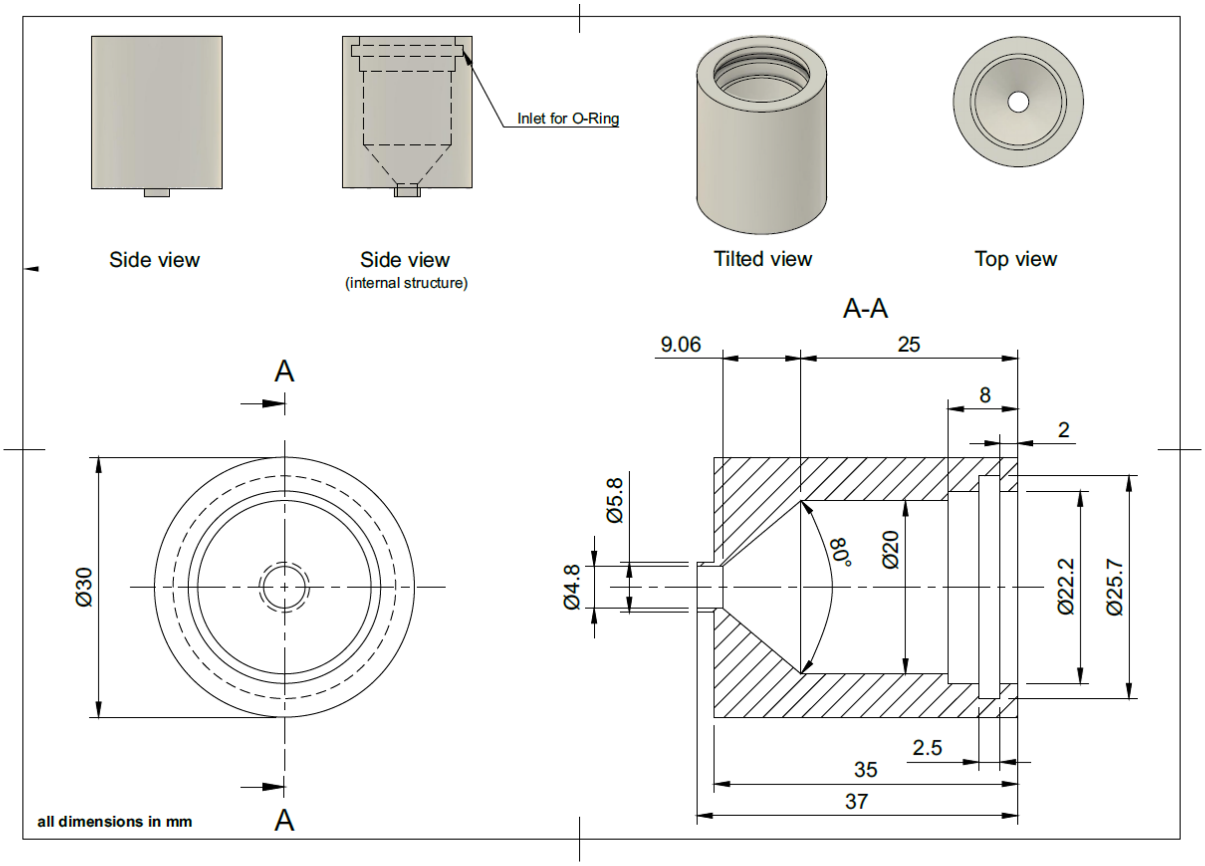

Supplementary Figure 4: Technical drawing of the deposition chamber. After the deposition chamber has been created, an O-Ring needs to be inserted into the inlet. This step is shown in the side view with the included internal structure. The files to 3D print the deposition chamber (.dwg/.stl), as well as the unscaled .pdf file of the technical drawing can be found in the download section of the supplementary information.

3 Perfusable platform “PerfuPul”

3.1 Design of the molds

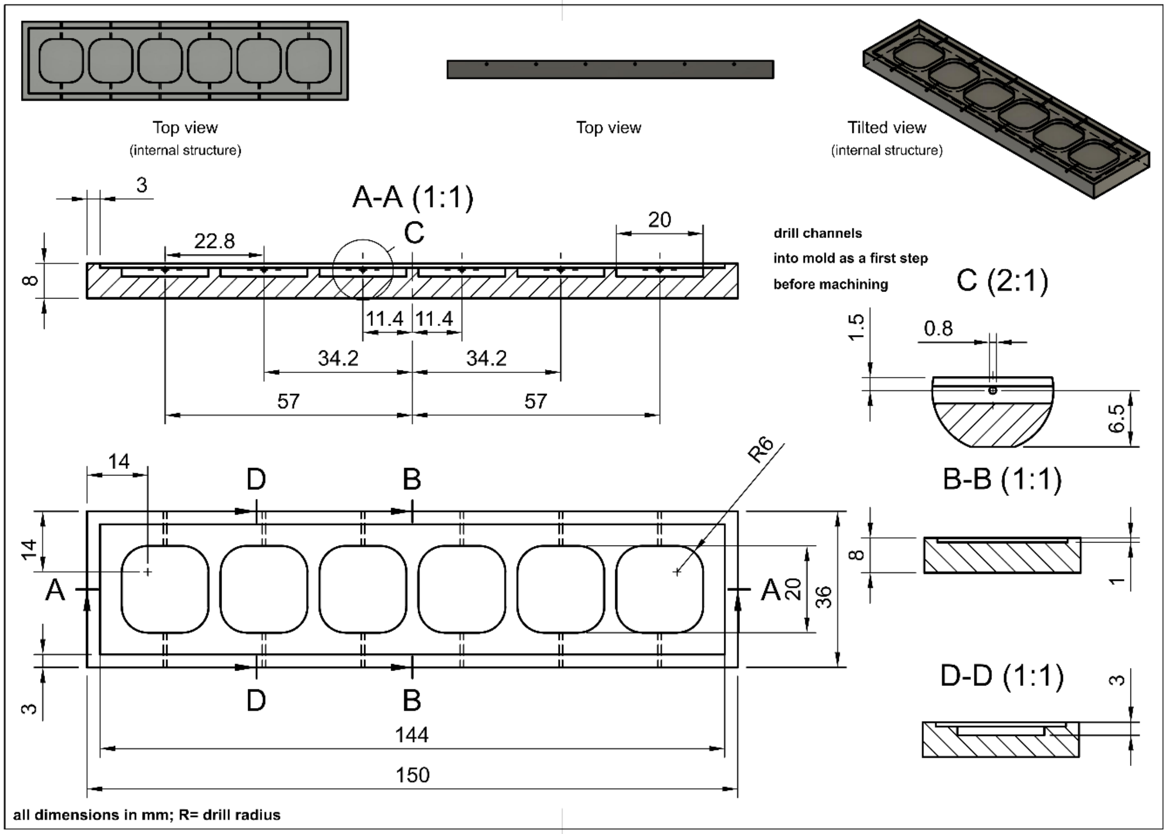

Supplementary Figure 5: Technical drawing of the mold (mold B) for creating the basolateral entities. It is important to note that the holes that later form the channels should be already drilled in the blank material, before the indentations are machined. In case of the mold to create the apical entities (mold A), the channel drilling step is just omitted. The files to 3D print the molds (A+B; .dwg/.stl), as well as the unscaled .pdf file of the technical drawing can be found in the download section of the supplementary information.

3.2 **PDMS preparation**

Materials needed: weighing dish (plastic), PDMS base + curing agent, desiccator, oven

1. Pre-heat oven to 100°C.
2. In a weighing dish add curing agent to Polydimethylsiloxane (PDMS) in a ratio of 1:10 (1:10 (w/w); curing agent/base) and mix thoroughly (> 2 min).
3. Carefully transfer mixed PDMS to a desiccator and degas. If big bubbles form during degassing (**Supplementary Figure** 6, left), ventilate and repeat desiccation process. Degas until PDMS-mixture displays a smooth air bubble free surface (**Supplementary Figure** 6, right) (~ 20 min).

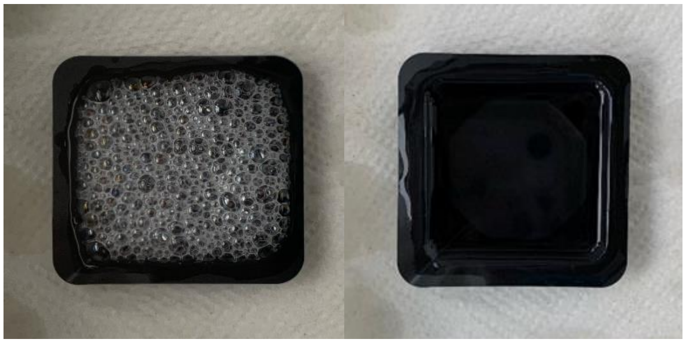

Supplementary Figure 6: Bubble creation during degassing of PDMS (left). After degassing a smooth bubble free surface should be obtained (right).

3.3 **Mold filling**

Materials needed: Needle (channel negative), mold A, mold B, oven, desiccator, freshly degassed PDMS (at least 25 g)

4. Insert eight needles (channel negative) in mold B that contains the inlets (Supplementary Figure 7, bottom). These entities will later form the basal part of the perfusable platform.

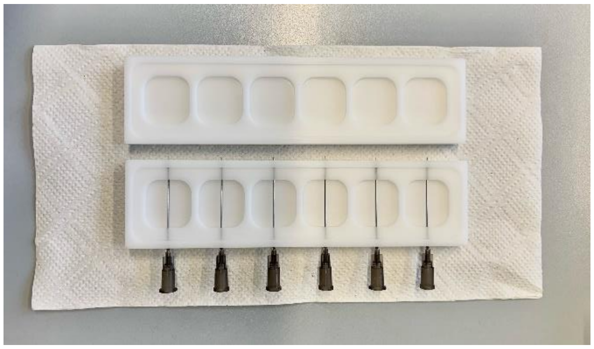

Supplementary Figure 7: top: mold A (designated for apical entities); bottom: mold B (designated for the basolateral entities). Use one needle to connect two perpendicular oriented channels.

5. Fill molds with freshly degassed PDMS using 8.5 g for mold B (basolateral entities, containing needles) and 9 g for mold A (apical entities).

6. After checking if molds are filled evenly and in their entirety, carefully transfer to desiccator and degas again until air bubble free (~ 20 min), opening the desiccator carefully if needed to insert air when strong bubble formation is observed.

7. Insert in preheated oven (100°C, 60 min).

8. Remove molds from oven and let them cool down completely before removing the previously inserted needles. Remove needles in a steady motion.

3.4 Entity production

Materials needed: scissors, biopsy punch, adhesive tape, blunt needle

9. Cautiously elevate the thin layer of polymerized PDMS surrounding the entities (Supplementary Figure 8, left) before carefully detaching it from the mold (Supplementary Figure 8, right).

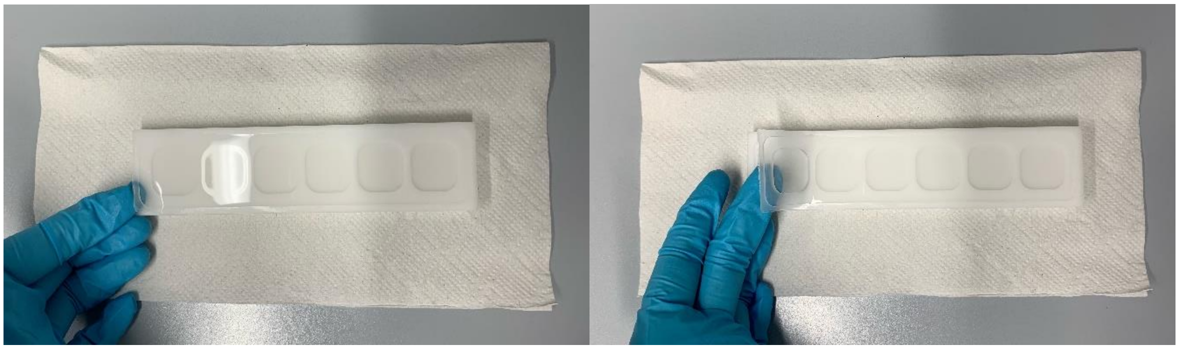

Supplementary Figure 8: Cautiously elevate the thin layer of polymerized PDMS surrounding the entities (a) before carefully detaching the PDMS from the mold (b).

10. Cut away protruding PDMS that surrounds the entities using scissors (Supplementary Figure 9).

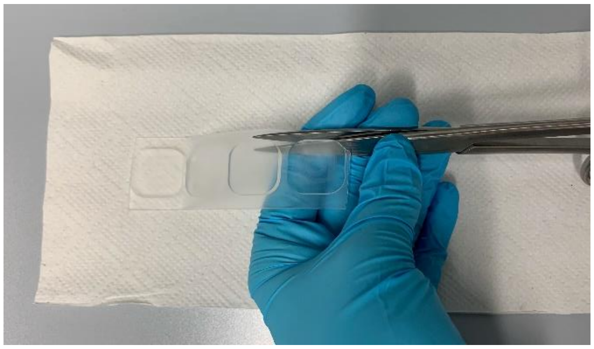

Supplementary Figure 9: Remove protruding PDMS that surrounds the entities with scissors.

11. Punch a centred hole using a 6 mm biopsy punch into the center of each entity.
12. Remove any PDMS residues that might block the channels of the basal entity with a blunt needle.
13. Use a stripe of adhesive tape to remove any leftover PDMS cuttings or dirt from the entities. Make sure to repeat until entity surfaces are clean.

3.5 Platform assembly

Materials needed: apical entities, basolateral entities, freshly degassed PDMS (at least 5 g), microscopy slide (76x52 mm), Transwells insert, scalpel, microscopy slides (24x32 mm), desiccator, oven, spin coater (if available)

Spin coater method (if no spin-coater is available, see step 16):

14. Clean microscopy slide (76x52 mm) (fits maximum 8 entities) by rinsing them with 100% Isopropanol first and afterwards with deionized water. Repeat this step twice. Check whether slides are clean, if not repeat. Dry slides using compressed air.

15. Cast a spot of freshly degassed PDMS (roughly 3 cm in diameter) onto one freshly dried microscopy slide (76x52 mm) and place it on a spin-coater (3000 rpm; 100 rpm/s; 60 s). Continue with **step 17**.

16. **Alternatively, in case no spin-coater is available**:

Add 150 mg of freshly degassed PDMS in-between two freshly cleaned microscope slides (step 14) and first apply firm pressure (

Supplementary Figure 10, left). Then slide them back and forth (

Supplementary Figure 10, center: left + right) until PDMS is well-distributed (

Supplementary Figure 10, right).

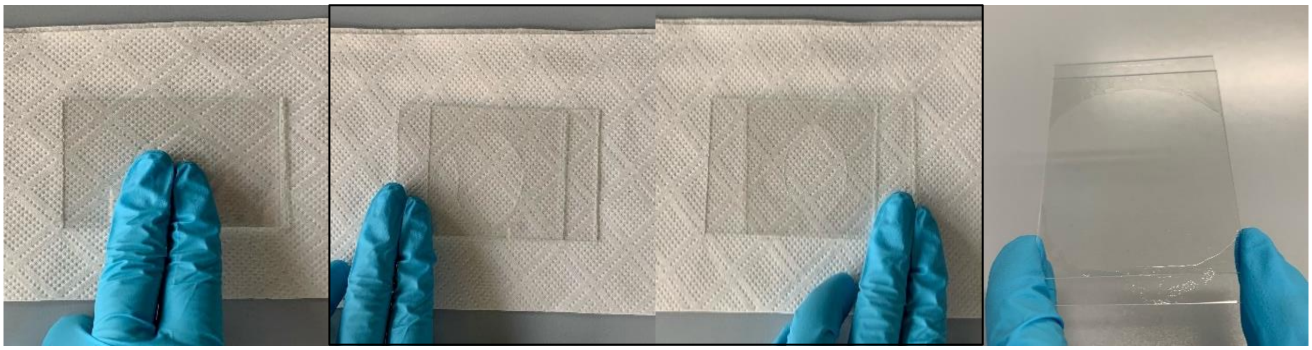

Supplementary Figure 10: First apply pressure on top of microscopy slides (76x52 mm) layered with PDMS (left), then slide them back and forth to distribute PDMS layered in-between (center: left + right). Apply firm but controlled pressure to ensure that the slides do not completely overlap and the PDMS is spread equally (right).

• Place microscopy slides (76x52 mm) in such a way into the desiccator that the slides do not overlap congruent. Degas in two intervals (2 x 10 min) and ventilate in-between. Air bubbles will not disappear completely but PDMS will be distributed more regularly (**Supplementary Figure 11**).

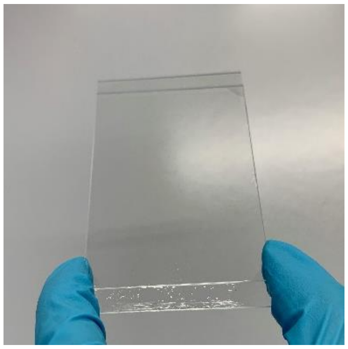

Supplementary Figure 11: More regular distributed PDMS-spot after first cycle of spreading and degassing**.**

• Remove microscopy slides (76x52 mm) from desiccator and separate them via a bilateral pulling motion, resulting in each of the separated slides coated with PDMS (**Supplementary Figure 12**, right). This step distributes the PDMS even more regular on both glass slides. Trapped air bubbles will also be eliminated, which can form unwanted streaks (**Supplementary Figure 13**).

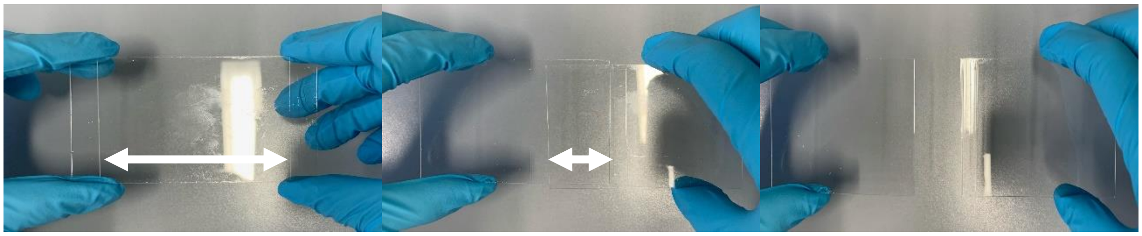

Supplementary Figure 12: Separation of microscopy slides (76 x 53 mm) by pulling them apart (left to right) in a steady bilateral pulling motion. (white arrows display the area where the slides overlap)

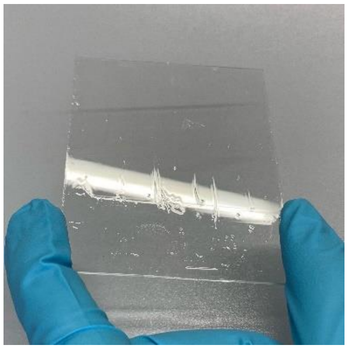

Supplementary Figure 13: Unwanted streaks formed during first separation.

- Place both microscopy slides (76x52 mm) congruent on top of each other and repeat sliding them back and forth as previously shown (**Supplementary Figure 12**). Afterwards degas and check whether unwanted streaks did form during separation (**Supplementary Figure 13**).

- If necessary, repeat until slides show a homogenous surface without any streaks (**Supplementary Figure 14**).

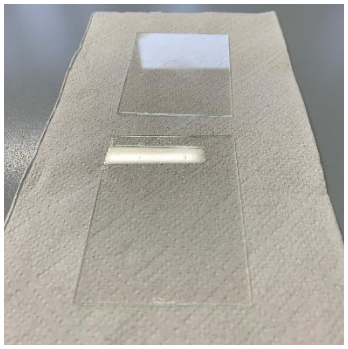

Supplementary Figure 14: Two microscopy slides (76x52 mm) covered with PDMS; no streaks, plain surface.

17. Place basolateral entity (resulting from mold B) onto one PDMS-covered microscopy slide (**Supplementary Figure 14**; fits 8 entities) and carefully apply firm pressure (**Supplementary Figure 15**, left). Avoid shifting entity. Then carefully lift entity from slide using index finger and thumb (**Supplementary Figure 15**, right).

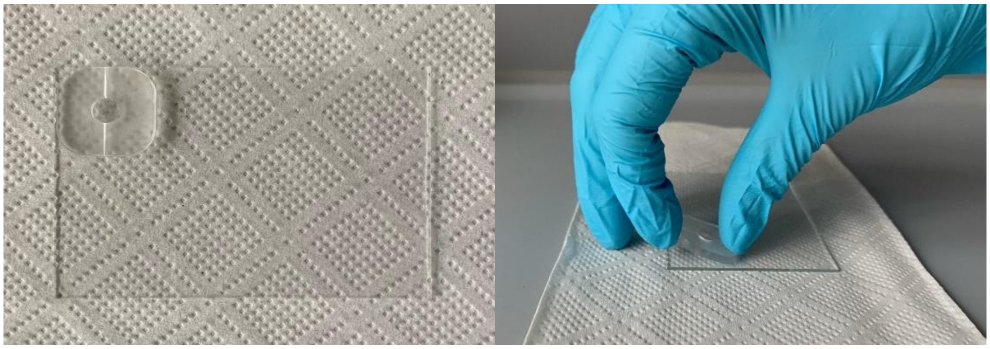

Supplementary Figure 15: First place basolateral entity on PDMS-covered microscopy slide (fits 8 entities, left) using firm pressure. Then use index finger and thumb to carefully lift PDMS-covered entity (right).

18. Attach PDMS-covered basolateral entities one after the other on a cover slide (24x32 mm). Pay attention to arrange channels in parallel to the short side of the microscopy slide. Apply pressure to ensure binding while simultaneously avoiding shifting the entities after placement. Be careful not to form new air bubbles after degassing.

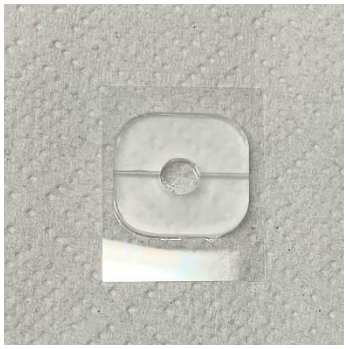

Supplementary Figure 16: Place basolateral entity onto microscopy slide, arrange channel in parallel to the short side of the microscopy slide. Be careful not to form new air bubbles after degassing.

19. Place all assemblies in desiccator and degas until air bubble free (10 min).

20. Carefully insert in preheated oven (100°C, 10 min).

21. Cover basolateral entities with adhesive tape and let cool.

22. Repeat **step 15** and coat apical entity (resulting from mold A) with PDMS. Alternatively use second microscopy slide from **step 16** if no spin-coater was available.

23. Place entity centrally on a Transwell insert so that the whole entity is in contact with the membrane area (Supplementary Figure **17**). Carefully apply pressure to ensure bonding. Avoid pushing entity around the membrane, as overflown PDMS might decrease the cell growth area (Supplementary Figure 18).

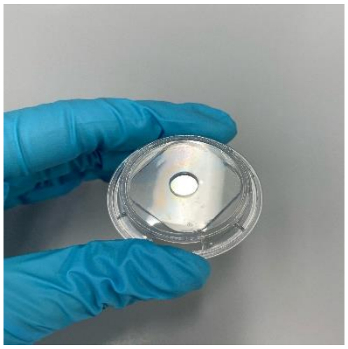

Supplementary Figure 17: Apical entity centrally placed on Transwell insert.

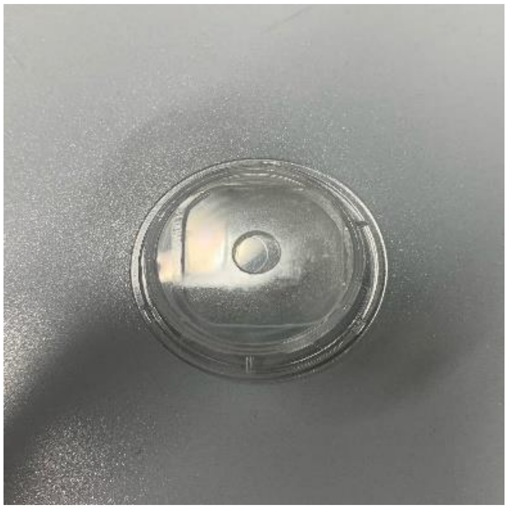

Figure 18: Reduced growth area due to unwanted entity movement after placed on transwell membrane.

24. Repeat for all apical entities.

25. Place assemblies in desiccator and degas until air bubble free (10 min).

26. Carefully insert assemblies in preheated oven (100°C, 10 min).

27. Cut entities from the Transwell insert using a scalpel (Supplementary Figure 19). Remaining air bubbles can be eliminated manually with a dull object, by carefully pushing against the membrane from the bottom side of the entity.

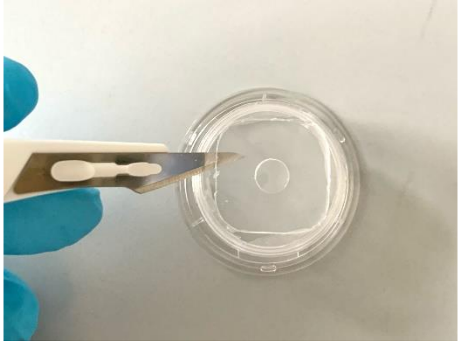

Supplementary Figure 19: Cut entity from Transwell membrane using a scalpel.

28. Repeat **step 15** (or alternatively **step 16** if no spin-coater is available) and apply PDMS onto basolateral entities from **step 21** (remove adhesive tape before applying PDMS).

29. Place the basolateral entity on membrane side of apical entity. Pay attention to align the pre-punched holes congruent.

30. Place the assembled platform in a desiccator and degas. Big air pockets may form during degassing in desiccator (Supplementary Figure 20). Fix it with PDMS in sealing step (step 31).

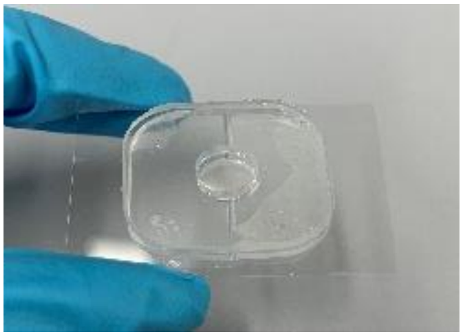

Supplementary Figure 20: Unwanted air pockets may form during degassing in desiccator. Fix it with PDMS in sealing step.

31. To remove any leftover air pockets, seal the platform. Therefore, carefully add liquid degassed PDMS (from step 15 or 16, or alternatively freshly mixed (~ 3 g)) on the edge that seals the basolateral and apical entity. Be careful not to put any PDMS in the openings of the channels.

32. Degas in desiccator (10 - 15 min). If needed repeat until there are no air bubbles left.

33. Place sealed platform in preheated oven (100°C, 10 min). The assembled perfusable platform should have the following characteristics: no air pockets, no reduced growth area as well as aligned wells.

3.6 Tube assembly

Materials needed: Tubing, scissors, blunt needle, spade, pliers, 2 ml reaction tube

34. Cut tubing into 10 cm fragments using scissors.

35. Isolate the metal cannula tube from a blunt needle using pliers. First crush the needle´s resin using pliers (Supplementary Figure 21, left). Be careful not to dent or crease the metal cannula tube. Then apply a firm use of pressure and repeatedly compress the resin to detach it from metal cannula tube (Supplementary Figure 21, center).

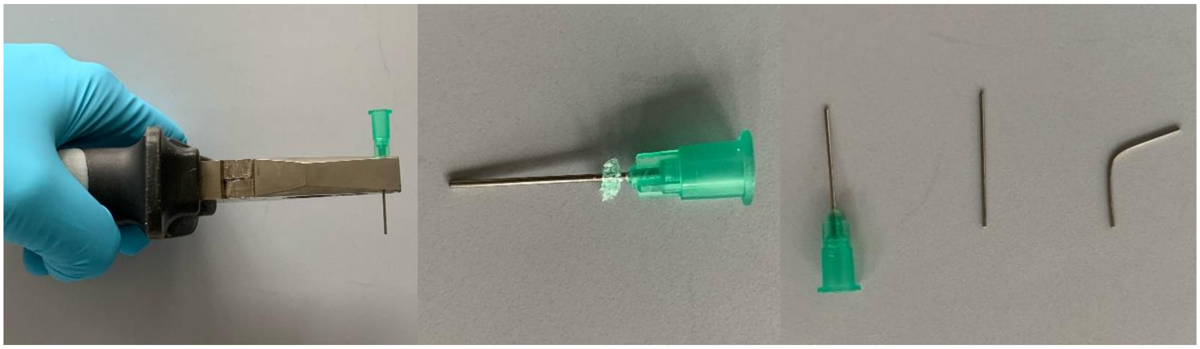

Supplementary Figure 21: Carefully crush the needle's resin using pliers (left); Resin successfully detached from metal cannula tube (center). Process of bending the connection piece for tube assembly: an isolated metal cannula tube is generated from a blunt needle. The cannula tube is bent around a 2 ml reaction tube to create the curvy connection piece (right).

36. Grab the plastic adhesive with pliers and use the other hand to pull on metal cannula tube. Disintegrate the needle in a steady motion.

37. Clean the isolated metal cannula tube of any resin residues. If necessary, use a laboratory spade.

38. Lastly take a 2 ml reaction tube and bend previously isolated metal cannula tube around to create a bow (Supplementary Figure 21, right). The curvy connection piece must not be pointy or sharp.

39. Each perfusable platform will need an inlet and outlet tubing: the inlet tubing has a curvy connection piece connected at one end and a blunt needle connected at the other end of the tubing (Supplementary Figure 22). The outlet tubing (not shown) is assembled leaving the blunt needle away.

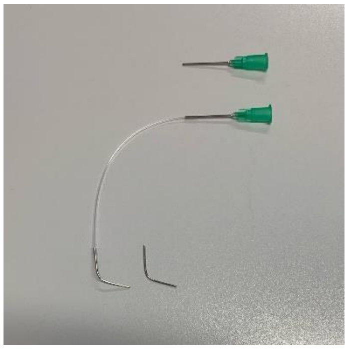

Supplementary Figure 22: Inlet tubing assembly as seen; Outlet tubing (not shown) re-assembled by connecting the curvy connection piece to a tubing, leaving one end of the tubing unmodified.

3.7 Preparing the perfusable platform for cell culture

To prepare the perfusable platforms for experiments that require sterilization such as cell culture, prepare: 1x petri dish, 1x assembled inlet tubing, 1x assembled outlet tubing, 1x tubing clamp, 1x autoclaved cover glass (∅ 12 mm) and 1x fully assembled platform per functional unit.

40. Immerse the inlet and outlet tubes as well as the tubing clamp for 10 min in 70% Isopropanol to disinfect them.

41. Put the material needed to assemble the functional unit (platform, inlet- and outlet tubes) into one petri dish each under a sterile lab hood (Supplementary Figure 23). Position the platform with the glass side facing up.

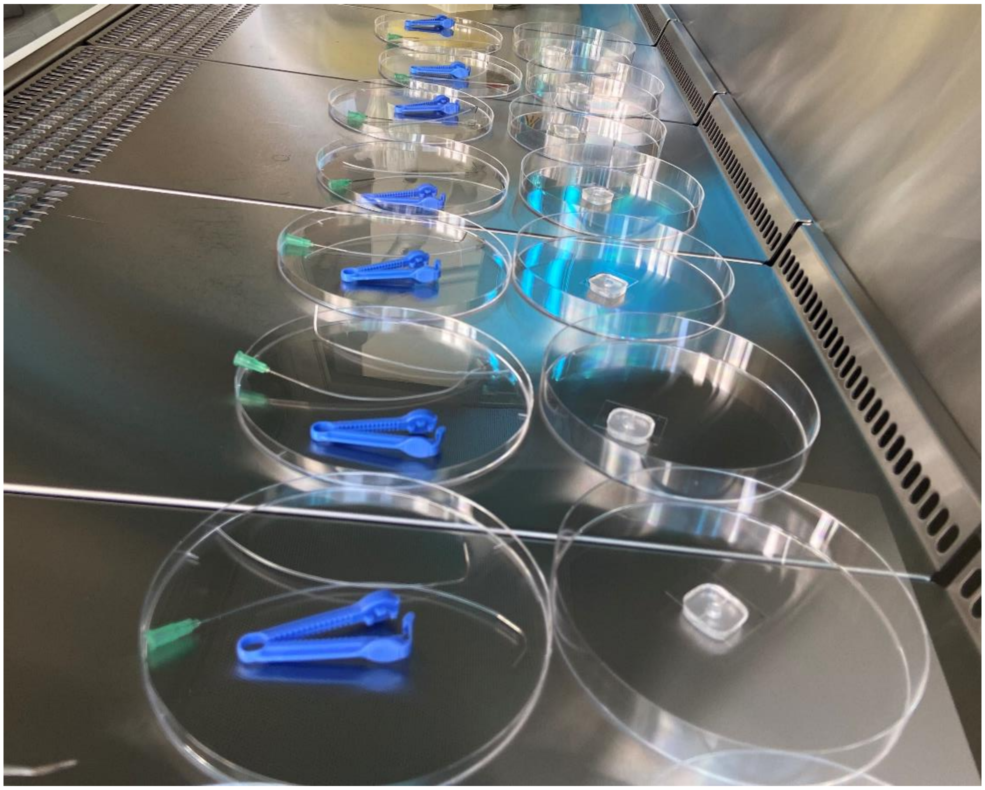

Supplementary Figure 23: Decontamination of functional unit.

42. Decontaminate the functional unit with UV light for 30 min.

43. Flip the platform as well as all other parts, except for the petri dish, towards the side that has not been in contact with UV.

44. Decontaminate again under UV light for 30 min.

45. Connect the tubes with the channel in- and outlet of the perfusable platform by inserting the curvy connection piece of each tubing into the channel opening. The fully assembled perfusable platform should look like the one displayed in Supplementary Figure 24. Close the platform with an autoclaved cover glass (∅ 12 mm) whenever needed.

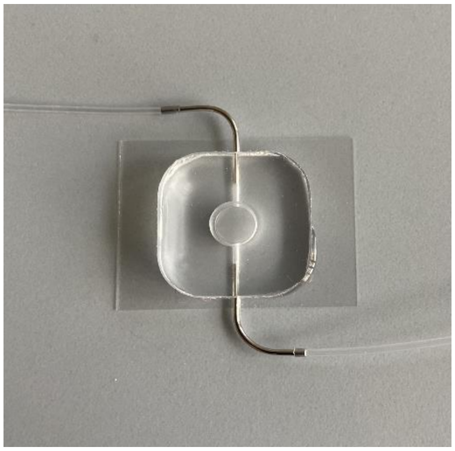

Supplementary Figure 24: Fully assembled and sealed perfusable platform. Characteristics: no air pockets, no reduced growth area as well as aligned wells.

46. Place all sterilized parts into the petri dish and close.

47. Please see the Material and Methods section of the main manuscript for details on cell culture.

3.8 Perfusion setup

Materials needed: 2x 15 ml reaction tubes (e.g. Falcon®), needle (channel negative) (step 4), 2x curvy connection pieces (step 38), 5 g freshly degassed PDMS (step 2+3), oven, sterile filter cap, tubing (step 34)

48. Punch a hole using a needle (channel negative) (step 4) at the 2 ml mark of one 15 ml reaction tube and also at the 14 ml mark of the other 15 ml reaction tube.

49. The 15 ml reaction tube with the hole at the 2 ml mark will be used as the inlet reservoir and the 15 ml reaction tube with the hole at the 14 ml mark will be used as the outlet reservoir.

50. Insert a curvy connection piece in each of the punched holes and place the curvy connection piece in such a way, that the curvy connection piece is stabilized by the inside of the reaction tube. Exemplarily shown in Supplementary Figure 25.

51. Seal the tubes with the inserted curvy connection pieces with freshly degassed PDMS from the inside as well as outside of the tube, put them in a desiccator, and degas for 15 min.

52. Insert the tubes in preheated oven (100°C) for 10 min.

53. Wash tubes with 10 ml distilled water.

54. Steam autoclave inlet and outlet reservoir with connected curvy connection pieces.

55. Close inlet and outlet reservoir with sterile filter cap under a sterile workbench.

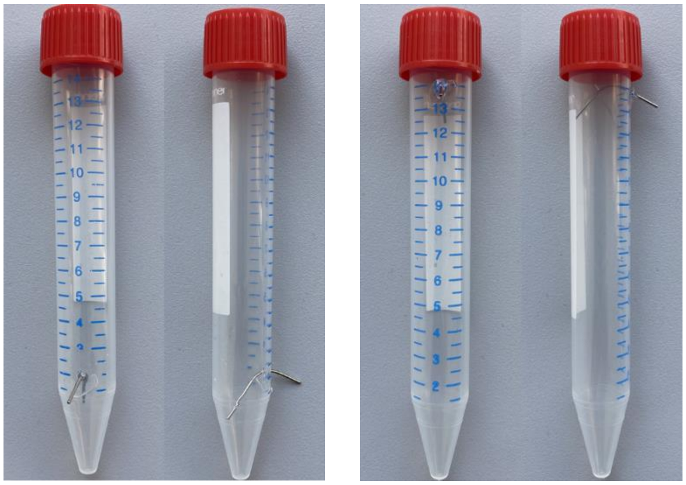

Supplementary Figure 25: Fully assembled inlet reservoir (left) and outlet reservoir (right) with sterile filter cap.

56. For perfusion experiments, connect the inlet reservoir to a peristaltic pump of your choice with tubing (step 34).

57. Fill the inlet reservoir with a liquid of your choice (e.g. cell culture medium) and flush all connecting tubing by using the peristaltic pump.

58. Once the tubing is cleared from any remaining air bubbles, connect a perfusable platform (fully assembled and sterilized) first to the peristaltic pump (which is connected to the inlet reservoir, step 57) and then to the outlet reservoir.

59. During the experiment watch that the liquid level in the inlet reservoir is in line with the membrane of the perfusable platform, to prevent an increase of hydrodynamic pressure.
