# Supplementary material for: PerfuPul—A Versatile Perfusable Platform to Assess Permeability and Barrier Function of Air Exposed Pulmonary Epithelia
Source: Front Bioeng Biotechnol. 2021 Oct 6;9:743236. doi: 10.3389/fbioe.2021.743236 (PMC8526933; doi:10.3389/fbioe.2021.743236)
Supplement: Supplementary file 2 [file DataSheet2.ZIP › Dimensions mold.pdf]

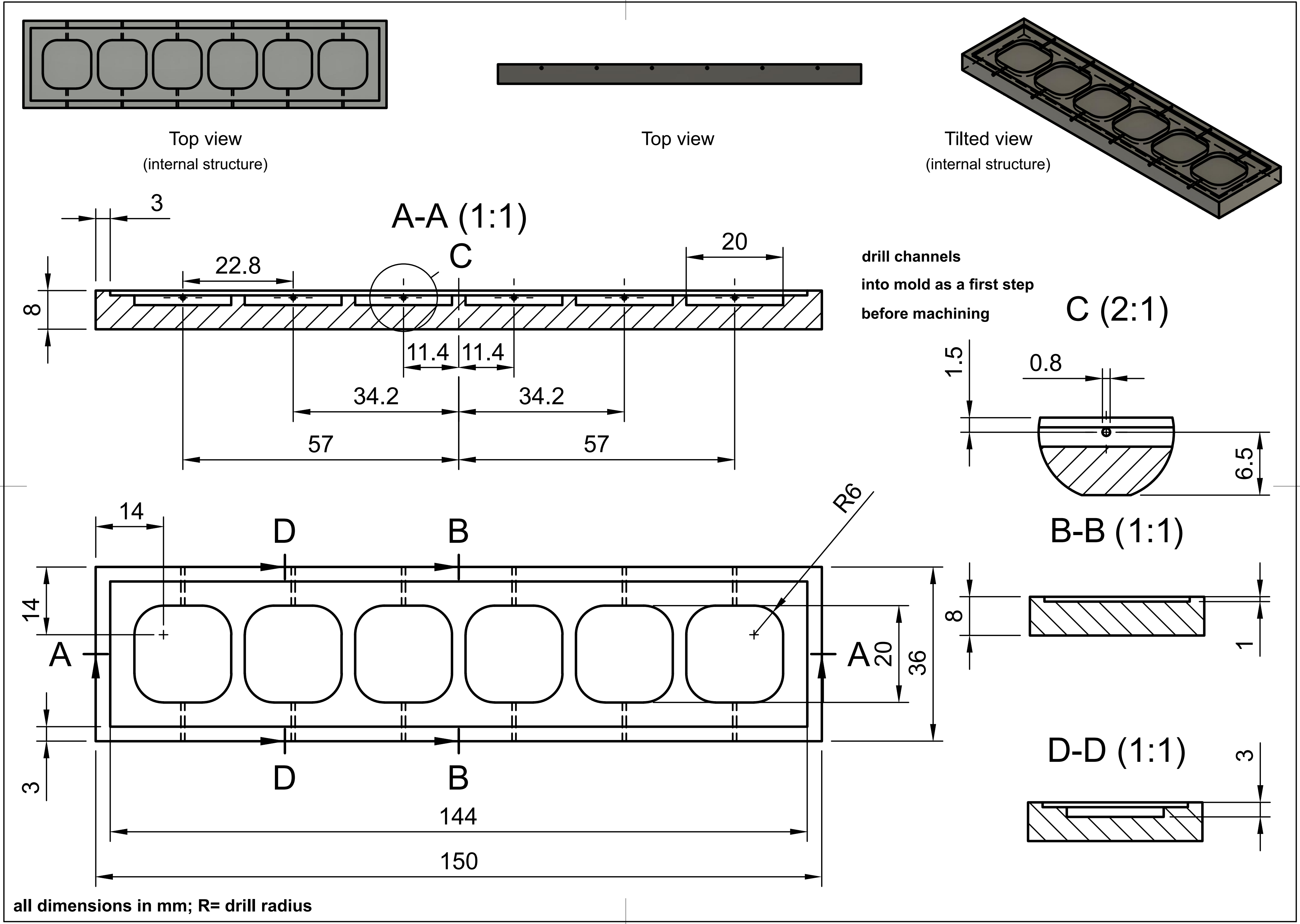

Top view
(internal structure)
Top view
Tilted view
(internal structure)
A-A (1:1)
3
22.8
C
20
8
11.4
11.4
34.2
34.2
57
57
drill channels
into mold as a first step
before machining
C (2:1)
1.5
0.8
6.5
R6
14
D
B
B-B (1:1)
14
A
A
20
36
8
1
3
D
B
D-D (1:1)
3
144
150
all dimensions in mm; R= drill radius
