# Supplementary figures and images for: PerfuPul—A Versatile Perfusable Platform to Assess Permeability and Barrier Function of Air Exposed Pulmonary Epithelia
Source: Front Bioeng Biotechnol. 2021 Oct 6;9:743236. doi: 10.3389/fbioe.2021.743236 (PMC8526933; doi:10.3389/fbioe.2021.743236)

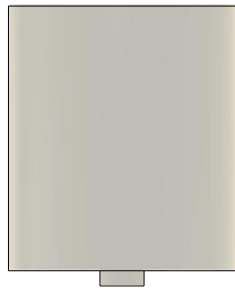

Side view

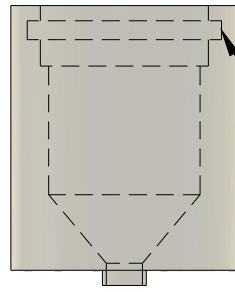

Inlet for O-Ring

Side view  
(internal structure)

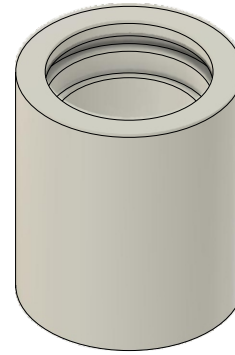

Tilted view

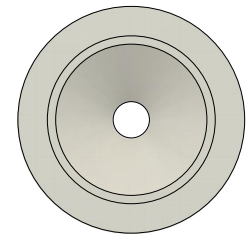

Top view

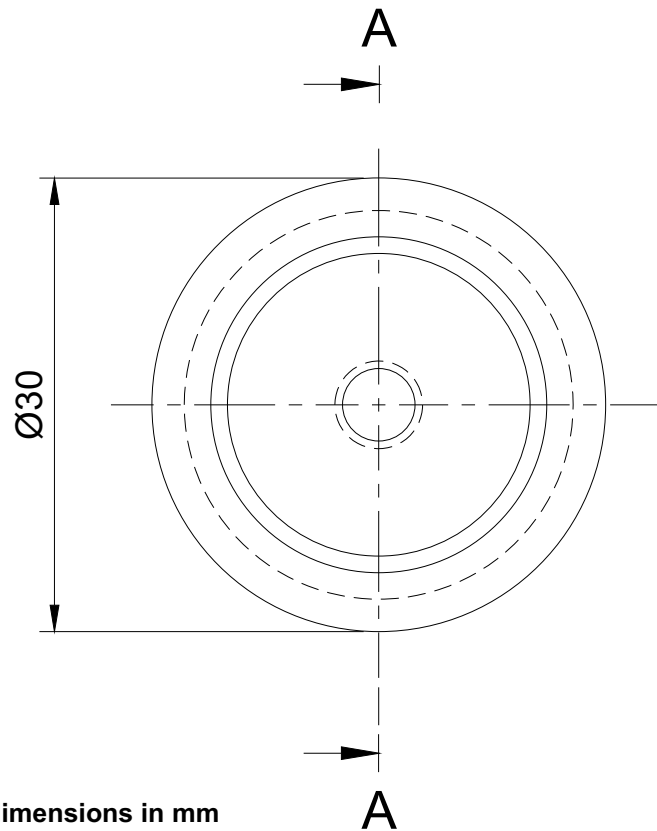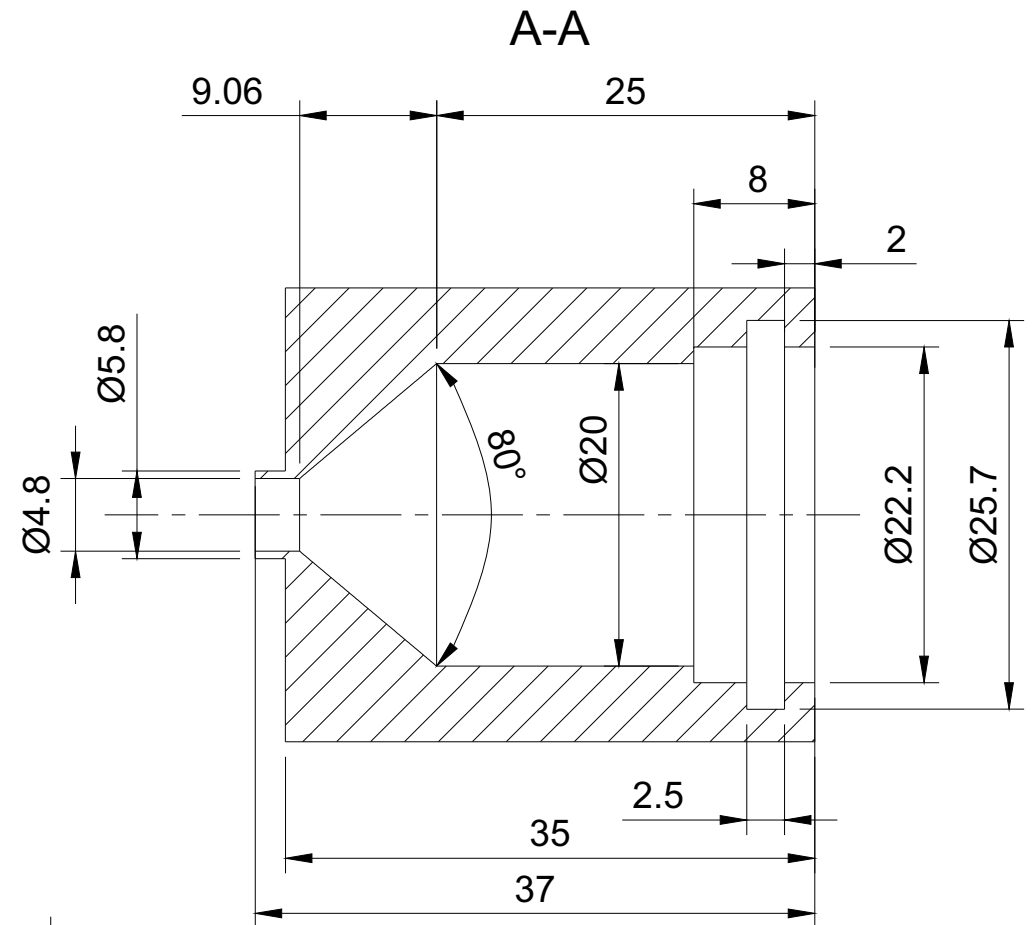

Supplement: Supplementary file 2 [file DataSheet2.ZIP › Dimensions deposition chamber.pdf]
